# Supplementary material for: Bulk-cusp microstructure for controllable multi-directional liquid spreading
Source: Nat Commun. 2026 Jan 8;17:1519. doi: 10.1038/s41467-025-68237-8 (PMC12913607; doi:10.1038/s41467-025-68237-8)
Supplement: Supplementary file 1 — Supplementary Information [file 41467_2025_68237_MOESM1_ESM.pdf]

Supporting Information

Bulk-cusp microstructure for controllable multi-directional liquid spreading

Songjie Dai [1], Hui Zhang [1,*], Yang Liu [1], Fenghao Yi [1], Kaibin Shi [1], Guangneng Dong [1]

[1] Key Laboratory of Education Ministry for Modern Design & Rotor-Bearing System, Xi'an Jiaotong University, Xi'an 710049, China

*Corresponding author: Hui Zhang (zhanghui7@xjtu.edu.cn)

The file includes:

- Supplementary Notes 1–4
- Supplementary Figs. S1–37
- Supplementary References 1–26

Supplementary Notes 1–4

Supplementary Note 1: Design strategy of the bulk-cusp microstructure

The cross-cusp and square-cusp microstructures are designed to regulate the self-driven spreading of the droplet body and precursor film, respectively, by precisely tailoring the orientation of the cusp structures. Each microstructure consists of a central bulk and directionally arranged cusps, which collectively dictate liquid spreading behavior. As shown in **Fig. S1a** and **Fig. S4a**, the microstructure bulk takes the form of either a cross or a square, with cusps systematically positioned along its 4 edges. The orientation of each cusp determines the spreading direction of the liquid. The cusp orientations are defined as left-forward (L-f), right-forward (R-f), left-backward (L-b), right-backward (R-b), front-left (F-l), front-right (F-r), back-left (B-l), and back-right (B-r). These directional elements govern the subsequent assembly of array units, giving rise to distinct liquid spreading behaviors.

As depicted in **Fig. S1b** and **Fig. S4b**, 4 individual microstructures (Individual I–IV) are constructed by varying the cusp orientations. Each unit exhibits a unique cusp arrangement, influencing liquid spreading in a predefined manner. Specifically, cusps positioned on the left and right sides dictate forward and backward spreading, whereas cusps located on the top and bottom modulate lateral transport. These fundamental structures serve as the building blocks for assembling arrays that enable controllable multi-directional liquid spreading. The transition from individual units to five distinct microstructure array patterns is illustrated in **Fig. S1c** and **Fig. S4c**. In the cross-cusp microstructure, spatial arrangement governs the spreading of the droplet body. The C-pinning mode confines the droplet at its initial position, preventing spreading. C-mode I introduces a unidirectional transport pathway, while C-mode II, C-mode III, and C-mode IV enable progressively more complex transport modes, facilitating bidirectional, tri-directional and quad-directional spreading. Similarly, for the square-cusp microstructure, different array configurations dictate precursor film spreading, where the S-pinning mode immobilizes the film, and S-mode I–IV progressively enable controlled spreading across 1 to 4 directions.

Supplementary Note 2: Quantitative characterization of anisotropic droplet spreading

Method 1 — Eight-direction length normalization and anisotropy factor

To compare spreading patterns generated by different microstructures, a direction-resolved metric is adopted that reports both how far the droplet spreads and how this extent is distributed angularly. The spreading length in a given direction is defined as the maximum projected distance from the droplet's geometric center to its outer contour, initially measured along the 4 Cartesian axes $x+$, $x-$, $y+$, $y-$. For cross-sample comparability, each directional length is normalized by the initial droplet radius R (the radius of the sphere droplet body), yielding normalized lengths L/R. A 4-axis description, however, cannot faithfully represent irregular spreading areas. The measurement is therefore extended to eight directions by adding 4 diagonals at 45°, 135°, 225°, and 315°. For any direction θ, the normalized length is:

$$\tilde{L}_{\theta} = \frac{L_{\theta}}{R},\ \theta \in \left\{x+,\ x-,\ y+,\ y-,\ 45^{\circ},\ 135^{\circ},\ 225^{\circ},\ 315^{\circ}\right\} \tag{S1}$$

The schematic of this measurement approach is provided in **Fig. S9a**, which visually demonstrates how directional lengths are extracted in both cardinal and diagonal directions. To better visualize how the eight-directional method is applied in practice, a set of schematic illustrations are constructed. As shown in **Fig. S9b**, this method captures the droplet's spreading front along both the Cartesian and diagonal axes, enabling improved angular resolution in quantification. Each panel depicts the geometric center of the droplet and the measured spreading lengths in eight directions, labeled as L_{x+}, L_{x-}, L_{y+}, L_{y-}, L_1, L_2, L_3, and L_4.

While this extension improves the fidelity of describing spreading profiles, a concise index is still required to quantitatively evaluate the degree of anisotropy. To this end, an anisotropy factor K is introduced based on the normalized lengths, enabling direct comparison of guided versus pinned behaviors. For Pinning and Mode IV (all-direction guidance), the overall extent of spreading is gauged by the average normalized radius:

$$K = \frac{L_{x+} + L_{x-} + L_{y+} + L_{y-}}{4R},\ \text{Pining mode or Mode IV} \tag{S2}$$

For Modes I–III, where guided and pinned directions coexist, K is constructed by comparing the guided axis with the three non-guided axes. The factor 3 balances the number of directions in the numerator and denominator:

$$K = \begin{cases} \dfrac{3L_{x+}}{L_{x-} + L_{y+} + L_{y-}}, \text{ Mode I} \\ \dfrac{L_{x+} + L_{y+}}{L_{x-} + L_{y-}}, \text{ Mode II} \\ \dfrac{L_{x+} + L_{x-} + L_{y+}}{3L_{y-}}, \text{ Mode III} \end{cases} \tag{S3}$$

Combined with the eight-direction normalization $\tilde{L}_\theta$, this scalar K provides a compact, reproducible, and geometry-tolerant quantification of multi-directional spreading that captures both magnitude and anisotropy.

Method 2 — Quadrant-based area of droplets and equivalent radius

To complement the direction-resolved length analysis, the second method is designed to quantify global spreading morphology in a geometry-tolerant manner. At the initial moment of contact, the droplet is approximated as a sphere with radius R, and the substrate is divided into 4 quadrants using a right-angle coordinate system centered at the droplet's geometric center. Each quadrant initially holds an area of:

$$S = \frac{1}{4}\pi R^2 \tag{S4}$$

After spreading, the droplet-covered area is segmented into quadrant areas S_1, S_2, S_3, S_4, which are normalized relative to S:

$$\tilde{S}_i = \frac{S_i}{S}, \text{ i} \in \{1,\ 2,\ 3,\ 4\} \tag{S5}$$

The total spreading area is defined as:

$$A_{\text{spread}} = S_1 + S_2 + S_3 + S_4 \tag{S6}$$

In addition, the total area can be used to define an equivalent spreading radius:

$$R' = \sqrt{\frac{A_{\text{spread}}}{\pi}} = \sqrt{\frac{S_1 + S_2 + S_3 + S_4}{\pi}} \tag{S7}$$

To better illustrate the quadrant-based area quantification strategy, a schematic diagram is presented in **Fig. S12a**. Upon spreading, the droplet-covered area is divided into 4 quadrants, each corresponding to an area S_1, S_2, S_3, S_4, which are normalized by the initial area defined by radius R. This visualization outlines how the total normalized spreading area and the equivalent spreading radius R' are derived, enabling a robust and geometry-tolerant quantification of non-axisymmetric spreading behaviour. To visualize the practical implementation of the quadrant-based area normalization method, representative steady-state imprints are extracted for various

structure–mode combinations. As shown in **Fig. S12b**, each panel displays the final spreading pattern segmented into 4 quadrant areas (S_1–S_4), cantered at the droplet's geometric centre. This method allows direct mapping of the spatial distribution of spreading into quantitative areal metrics, thereby facilitating standardized, geometry-independent comparisons across diverse structural designs and spreading modes.

Supplementary Note 3: Mechanical analysis of multi-directional liquid spreading and pinning

To address the complexity of force interactions during liquid spreading, the transient spreading state of the liquid interface is idealized for analysis. It is assumed that the contact angle between the precursor film and both the sidewalls and bottom surface is equal to the intrinsic contact angle. The precursor film is considered to fully fill the bulk-cusp microstructure, with the contact line on the sidewalls forming a continuous inclined line. As illustrated in **Fig. S30a**, the capillary attraction force from the sidewall $F_{\gamma l}$, tangent to the liquid interface and pointing outward, is given by **Equation (S8)**:

$$F_{\gamma l} = \gamma \cdot \frac{h}{\sin\theta} \tag{S8}$$

where γ is the surface tension between the liquid and the hydrophilic walls, h is the depth of the microstructure, and θ is the intrinsic contact angle. Similarly, the bottom surface generates a lateral capillary attraction $F_{\gamma s}$, given by **Equation (9)**:

$$F_{\gamma s} = \gamma \cdot l \tag{S9}$$

where l is the local width of the precursor film at the cusp (**Fig. 3c**). These forces are decomposed in the x–y plane to yield components F_1 and F_2 (**Equation (S10–S11)**):

$$F_1 = F_{\gamma l} \cdot \sin\theta = \gamma \cdot h \tag{S10}$$

$$F_2 = F_{\gamma l} \cdot \cos\theta \cdot \sin\theta = \gamma \cdot h \cdot \cos\theta \tag{S11}$$

Further decomposition along the x-direction yields the capillary driving force F_{d1} and backward resistance F_{b} generated by the sidewalls (**Equation (S12–S13)**):

$$F_{\mathrm{d1}} = 2 \cdot F_2 \cdot \cos(\alpha - \beta) \tag{S12}$$

$$F_{\mathrm{b}} = 2 \cdot F_1 \cdot \sin(\alpha - \beta) \tag{S13}$$

where α and β are the angle between the cusp and bulk structure, and the apex angle of the cusp, respectively.

The capillary attraction from the bottom surface F_{d2} is given by **Equation (S14)**:

$$F_{\mathrm{d2}} = F_{\gamma s} \cdot \cos\theta = \gamma \cdot l \cdot \cos\theta \tag{S14}$$

The total forward capillary driving force F_{d} and resistance F_{b} are thus (**Equation (S15–S16)**):

$$F_{\mathrm{d}} = 2 \cdot \gamma \cdot h \cdot \cos\theta \cdot \cos(\alpha - \beta) + \gamma \cdot l \cdot \cos\theta \tag{S15}$$

$$F_{\mathrm{b}} = 2 \cdot \gamma \cdot h \cdot \sin(\alpha - \beta) \tag{S16}$$

Accordingly, the resultant force F acting on the precursor film in the forward direction is given by **Equation (S17)**:

$$F = F_{\mathrm{d}} - F_{\mathrm{b}} = 2\gamma h\cos\theta\cos(\alpha-\beta) + \gamma l\cos\theta - 2\gamma h\sin(\alpha-\beta) \tag{S17}$$

For precursor film forward spreading, $F > 0$ should be satisfied. When $90° \le \theta < 180°$, corresponding to hydrophobic or superhydrophobic surfaces, $F = F_{\mathrm{d}} - F_{\mathrm{b}} < 0$, and spreading is inhibited. When $0 < \theta < 90°$, the necessary condition for spreading holds (**Inequation (S18)**):

$$\cos\theta > \frac{2h\sin(\alpha-\beta)}{l + 2h\cos(\alpha-\beta)} \tag{S18}$$

Equation (S17) shows that F is positively correlated with l. Given that the minimal width between cusps is W=3 μm and h=12 μm, the directional spreading condition simplifies to **(Inequation (S19))**:

$$\cos\theta > \frac{8\sin(\alpha-\beta)}{1 + 8\cos(\alpha-\beta)} \tag{S19}$$

Accordingly, the relationship between θ and (α–β) defines the feasible design space for forward spreading, as illustrated by the shaded region in **Fig. 3d**. The relation demonstrates a negative correlation between force magnitudes and both θ and (α–β), implying that smaller values of θ and (α–β) enhance spontaneous liquid spreading. Points closer to the origin represent greater driving forces. The red dot in **Fig. 3d** marks the parameter set used in this study, confirming the suitability of the selected geometry for guided precursor film spreading.

In addition to promoting forward spreading, the sharp edges of the cusp structure provide effective pinning in the reverse direction due to the sharp edge effect, as shown in **Fig. 3f–g.** When a droplet contacts a sharp edge with an inclination angle φ smaller than the intrinsic contact angle θ, the real contact angle becomes larger than the nominal one, inhibiting spreading or collapse. The critical contact angle θ_c at the cusp edge can be approximated by (**Equation (S20)**):

$$\theta_c = (180° - \varphi) + \theta \tag{S20}$$

Thus, sharp cusp edges hinder reverse spreading of the droplet body. As illustrated in **Fig. 3e**, the pinning resistance is analyzed by resolving the capillary forces along the sidewalls and bottom substrate. The liquid surface at the instant of pinning is assumed to be cylindrical for simplicity. When $\alpha \le \theta < 90°$, the capillary force F_{b}' exerted by the inclined sidewalls on the precursor film can be expressed as **Equation (S21)**:

$$F_{\mathrm{b}}' = 2\gamma h\cos(\theta-\alpha) \tag{S21}$$

Meanwhile, the partial contact of the liquid film with the bottom surface contributes an

opposing force in the horizontal direction, given by **Equation (S22)**:

$$F_{\mathrm{d2}}' = \gamma l \tag{S22}$$

The resultant capillary resistance acting against reverse motion is therefore the difference between these two components, given by **Equation (S23)**:

$$F' = F_{\mathrm{b}}' - F_{\mathrm{d2}}' = 2\gamma h \cos(\theta - \alpha) - \gamma l \tag{S23}$$

When $\theta < \alpha$, the capillary interaction along the sidewall is reduced. However, the bottom surface still contributes an opposing horizontal component. The total resistance force is then expressed as **Equation (S24)**:

$$F' = F_{\mathrm{b}}' - F_{\mathrm{d2}}' = 2\gamma h \cos(\alpha - \theta) - \gamma l \tag{S24}$$

To ensure effective backward pinning, the net resistance must satisfy $F' > 0$. For the case $\alpha \le \theta < 90°$, this yields the geometric constraint, given by **Inequation (S25)**:

$$\cos(\theta - \alpha) > \frac{l}{2h} \tag{S25}$$

Inequation (S25) provides the geometric constraint for achieving backward pinning. Given that the minimal width between cusps is *W*=3 μm and *h*=12 μm, the ratio simplifies to $\frac{l}{2h} = \frac{1}{8}$.

Substituting this into **Inequation (S25)** yields the simplified constraint, given by **Inequation (S26)**:

$$\cos(\theta - \alpha) > \frac{1}{8} \tag{S26}$$

This inequality defines the necessary condition for effective pinning of the precursor film by the cusp structure. As illustrated in **Fig. S30b**, identifies the geometric combinations of θ and α satisfying the constraint **Inequation (S26)**, which ensures sufficient backward pinning. For the intrinsic contact angle θ=35°, the critical threshold is approximately α<117.8°. The parameter points corresponding to the designed cusp geometry and the reference line of $\alpha = \theta$ are also marked, indicating that points closer to the $\alpha = \theta$ line experience stronger resultant pinning forces, thus enhancing the backward resistance effect.

Supplementary Note 4: Benchmarking against existing literature with quantitative metrics

Based on quantitative metrics to support the performance improvements, we provide a more comprehensive comparison with existing literature from the perspective of both lubrication and thermal management applications.

In our study, the cross-cusp microstructures positioned in the non-contact region created continuous capillary pathways that passively delivered lubricant into the interface. This mechanism maintained a stable friction coefficient of about 0.2, while the bare surface rose to about 0.3, yielding an overall reduction of about 35%. Such performance demonstrates that the proposed design can achieve substantial friction reduction without any external energy supply, while also ensuring long-term stability under dynamic conditions. In recent years, researchers have explored a variety of advanced strategies to reduce friction, which can generally be classified into 4 categories: protective or functional coatings, lubricant additives, surface texturing within the contact zone, and surface strengthening or modification techniques. Considerable efforts have been devoted to each of these approaches, and their current status is summarized below to provide a clear benchmark for comparison.

(1) **Protective or functional coatings:** Coating technologies have recently attracted considerable attention as an effective means of reducing friction. Jin et al. reported that a porous textured DLC/MAO multilayer coating markedly lowered the friction coefficient under dry and oil-lubricated conditions, with about 20.9% reduction achieved in the latter[1]. Lin et al. showed that chrome plating on polymer–metal friction pairs decreased the friction coefficient by around 20%, owing to improved hardness and the formation of a stable transfer film[2].

(2) **Lubricant additives:** As a complementary path to coatings, lubricant additives provide a mature route to friction reduction with clear quantitative benchmarks. Dou et al. demonstrated that self-dispersed crumpled graphene balls in base oil lowered the friction coefficient by about 20% compared with pure oil, showing superior dispersion stability and tribological performance[3]. Hou et al. prepared graphene additives for low-sulfur diesel via plasma-assisted ball milling and reported a 20–24% reduction in the friction coefficient depending on the fuel type[4]. Karimi et al. showed that adding 0.2 wt% CuO nanoparticles to HB-80 turbine oil lowered the friction coefficient by 22.86%[5]. Kumar et al. investigated h-BN nanoadditives in aerospace-grade lubricants and found that the addition of 0.1 wt% h-BN reduced the friction coefficient by 13% while enhancing lubricant stability[6].

(3) **Surface texturing directly within the contact zone:** Surface texturing of the contact zone has also been proven effective in lowering friction. Liu et al. investigated laser-induced circular dimples, and the textured surfaces exhibited up to a 27% lower friction coefficient compared with smooth counterparts[7]. In another study, Cao et al. examined cylindrical-ground microstructural surfaces and found that, under well-lubricated conditions, optimized textures improved friction performance by up to about 20% versus smoother, untextured counterparts[8]. Chen et al. enhanced the tribological performance of cylinder guide bushes by introducing bionic convex textures with composite grease, resulting in a 5.0–6.7% reduction in friction coefficient[9]. Ge et al. used multivariate linear regression to optimize micro-texture parameters and reported a 15.6% reduction in friction coefficient compared with texture-free surfaces[10].

(4) **Surface strengthening or modification techniques:** Beyond coatings, additives, and in-contact texturing, other surface strengthening routes have also delivered measurable friction reduction. Cai et al. applied shot peening to CF53 steel and reported a 22.5% decrease in the friction coefficient relative to untreated samples[11]. Zhang et al. strengthened GCr15 spherical joint bearings via ultrasonic surface rolling, achieving a 28% reduction in the friction coefficient[12]. Hu et al. introduced low-frequency vibration during laser wire additive manufacturing of thin-walled Inconel 601, lowering friction coefficients by 12.6% (dry)–14.2% (oil)[13]. Acharya et al. used surface mechanical attrition treatment on a Ti–Nb–Ta–O alloy and, in the β+α aged condition, observed a 21% reduction in friction coefficient under fretting[14].

From the above literature review, it can be concluded that the reduction rates achieved by currently advanced strategies, including coatings, lubricant additives, surface texturing, and other strengthening techniques, are consistently below 30% in the available reports. In contrast, as illustrated in **Fig. S35**, our proposed method enables a friction coefficient reduction of about 35%, which is higher than those achieved by the existing approaches. The superior performance originates from the cross-cusp microstructures located outside the contact region (no wear occurs), which passively and continuously replenish lubricant into the interface without the need for external energy. This design not only stabilizes the friction coefficient over time but also ensures long-term effectiveness under varying operating conditions. Therefore, the strategy is not merely an incremental improvement but represents an innovative route that couples structural design with passive capillary-driven transport, offering a novel and energy-efficient pathway for advanced lubrication management.

In terms of thermal management, our work introduces a novel square-cusp microstructure that enables the directional spreading of a precursor film while leaving the droplet body almost stationary. This design ensures a continuous supply of precursor liquid that spreads outward in a sustained manner, thereby realizing efficient cooling of the heated substrate through evaporation. Such a mechanism represents a conceptual innovation with strong potential for applications in intelligent thermal regulation of electronic chips and other advanced devices. Importantly, it not only guarantees sustainable liquid supply but also offers programmable, directional, and intelligent control of heat dissipation, setting it apart from existing passive cooling approaches. At present, however, there are no directly comparable studies in the literature that provide quantitative benchmarks equivalent to our results. Instead, research in advanced thermal management has mainly progressed along several directions, such as surface modification to enhance evaporation, nanostructured coatings for improved heat transfer, capillary-driven wicking structures, and hybrid systems coupling photothermal or electrohydrodynamic effects. The following section summarizes and compares these representative strategies to provide a broader context for positioning our method.

Recent advances in thermal management have followed 4 main routes:

(1) Surface modification to enhance evaporation: Berce et al. investigated laser-textured copper with tuned wettability to manage nanoparticle deposition and sustain boiling performance on structured heaters[15]. Orman et al. investigated laser-generated grooves and microfins on copper, showing how geometry and roughness tailor nucleation and film dynamics in pool boiling[16]. Orman et al. further investigated laser process parameters (pulse duration/scan speed) to engineer multi-scale topographies that improve pool-boiling heat transfer[17].

(2) Nanostructured coatings to improve heat transfer: Zhao et al. investigated ceramic-coated carbon nanotube (CNT) microstructures (via atomic layer deposition (ALD)) to create mechanically stable micro/nanoporous boiling interfaces with rapid liquid imbibition[18]. Sen et al. investigated Cu–TiO_2 nanoparticle coatings prepared by a hybrid method on copper to alter porosity and surface energy for enhanced pool boiling[19]. Kumar et al. investigated two-step electrodeposited Cu–TiO_2 nanocomposite coatings to study how deposition strategy controls coating integrity and boiling behavior[20].

(3) Capillary-wicking liquid-transport structures: Chun et al. investigated hierarchical copper nanowire arrays with interconnected V-grooves to accelerate thin-film wicking for electronics cooling[21]. Luo et al. investigated biomimetic "copper-forest" wicks for ultrathin heat pipes/vapor chambers to strengthen capillary lift in compact spreaders[22]. Luo et al.

also investigated copper-mesh wicks modified with a copper-forest structure to balance permeability and capillary pressure[23].

(4) Hybrid systems coupling photothermal or electrohydrodynamic effects: Wang et al. investigated graphene-assisted ionic-wind cooling for LEDs to intensify corona discharge and airflow over hot components[24]. Xu et al. investigated a tri-needle/ring ionic-wind device embedded in LED bulbs to enhance forced convection around filaments[25]. Cheng et al. investigated electrohydrodynamic gas pumps with aligned/offset electrodes for channel flows in electronics cooling[26].

In comparison, these 4 routes largely i) operate in the contact/heated zone by altering nucleation and surface energy (surface modification, nanocoatings), ii) rely on sealed two-phase loops with designated wicks and working liquids to spread and return liquid (capillary structures), or iii) require external energy or fields to drive flow and heat removal (photothermal/EHD). By contrast, our square-cusp microstructure routes a precursor film from the droplet body a stable source while a thin film is continuously and directionally delivered to the hotspot, enabling sustained supply and programmable, anisotropic evaporative cooling without complex external devices, such as pumps. This non-contact, lithography-defined routing offers a distinct, energy-free pathway suited to intelligent, layout-aware heat management at device scale.

Supplementary Fig. S1–37

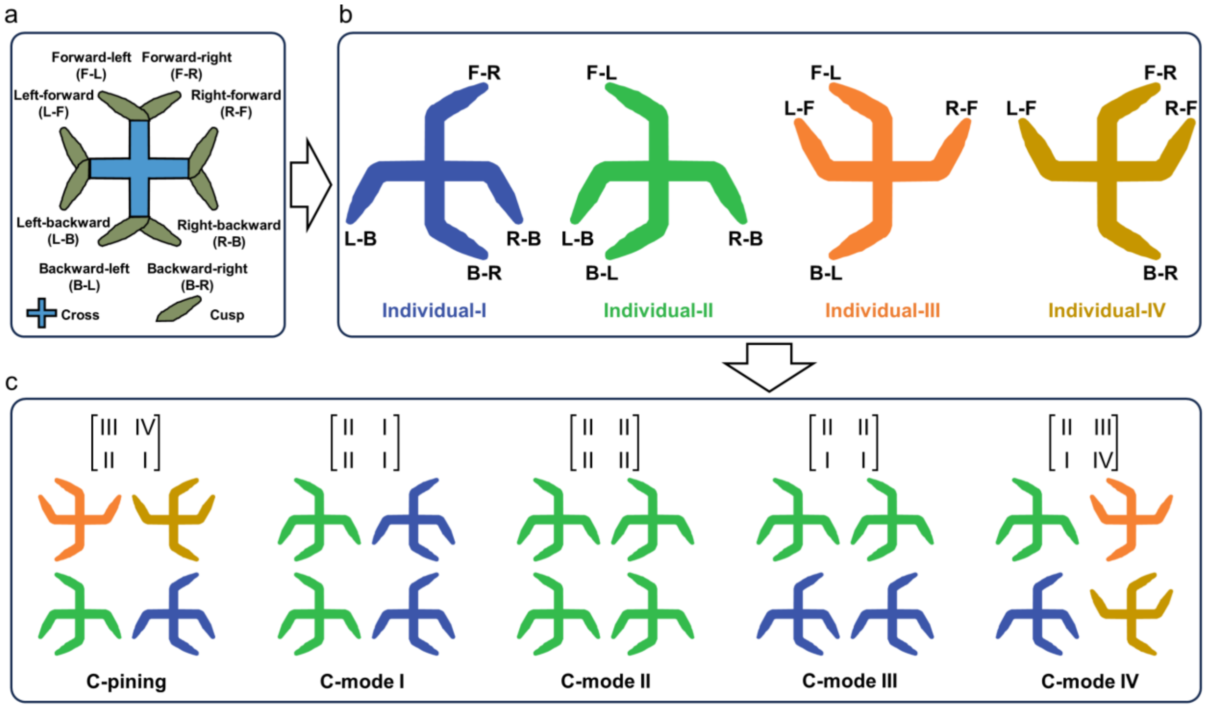

Fig. S1. Design of the cross-cusp microstructure and its liquid spreading patterns. a Schematic illustration of the cross-cusp microstructure, showing the integration of a cross-shaped bulk with directionally arranged cusps. **b** Four distinct microstructure individuals constructed by varying the cusp orientations along the bulk edges. **c** Five array patterns formed by systematically arranging the individuals, enabling liquid pinning (C-pinning), unidirectional (C-mode I), bidirectional (C-mode II), tri-directional (C-mode III), and quad-directional (C-mode IV) spreading.

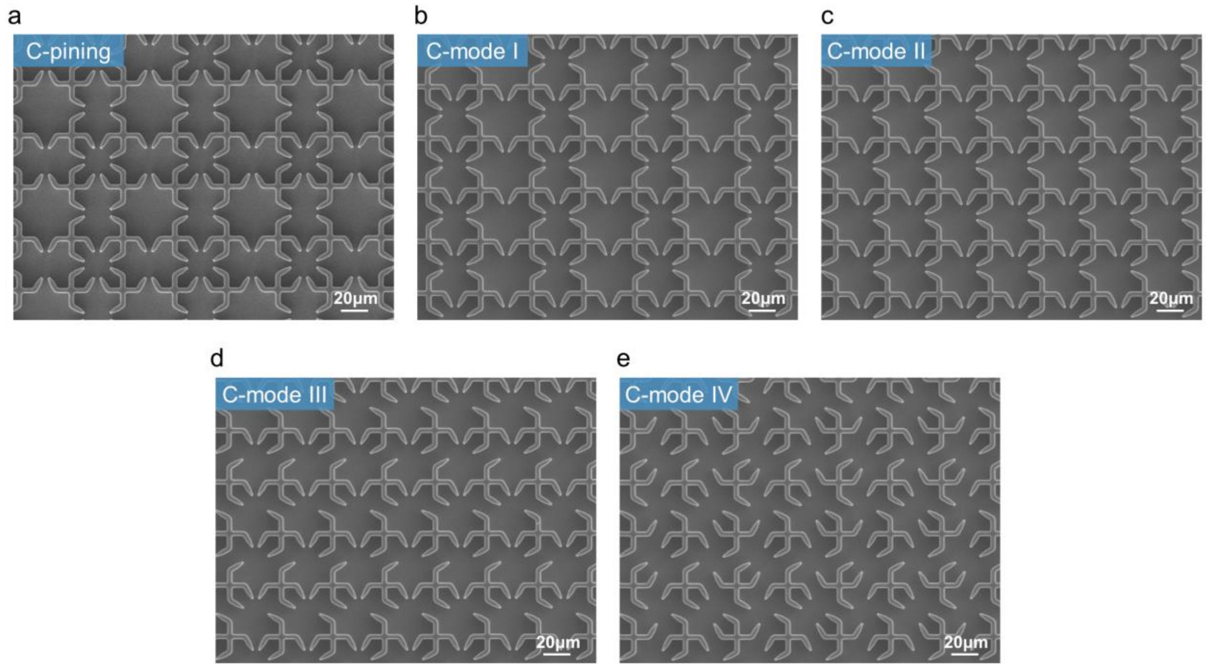

Fig. S2. SEM images of the cross-cusp microstructure exhibiting different liquid spreading modes. a C-pinning mode, where the droplet remains pinned without noticeable spreading. **b** C-mode I, where the droplet body and precursor film spread unidirectionally along a predefined path. **c** C-mode II, enabling bidirectional spreading along two directions. **d** C-mode III, where the droplet body and precursor film expand in three directions. **e** C-mode IV, facilitating quad-directional spreading.

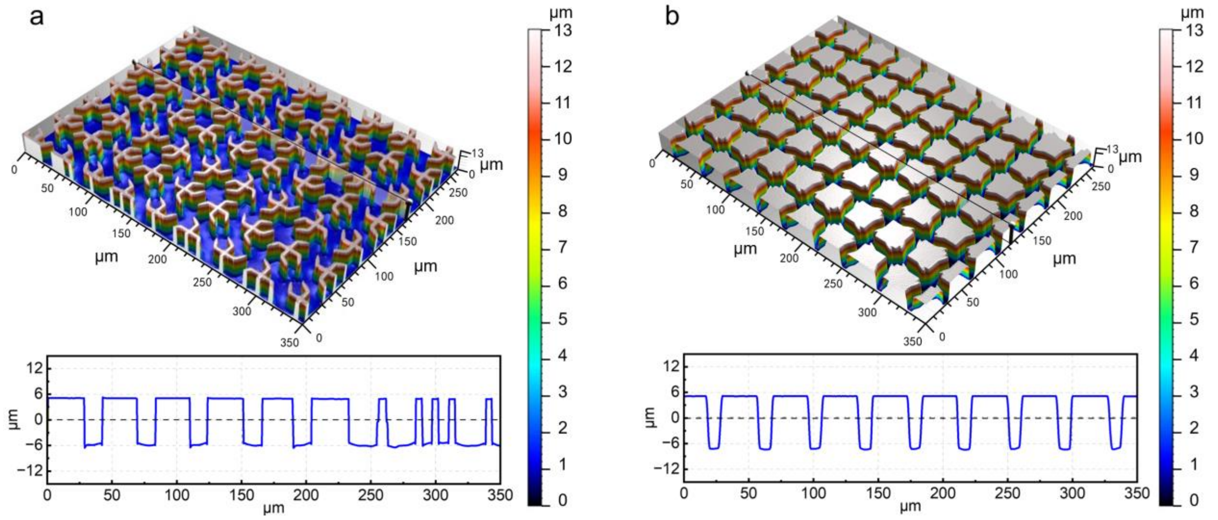

Fig. S3. Three-dimensional morphology and cross-sectional profiles of cross-cusp and square-cusp microstructures. a 3D topographic reconstruction of the cross-cusp microstructure (C-mode IV), highlighting the cross-shaped body with cusps, while the cross-sectional profile indicates a uniform structural depth of approximately 12 μm. **b** 3D topographic reconstruction of the square-cusp microstructure (S-mode IV), featuring the square-shaped body with cusps, while the cross-sectional profile reveals a consistent structural depth of approximately 12 μm. Source data are provided as a Source Data file.

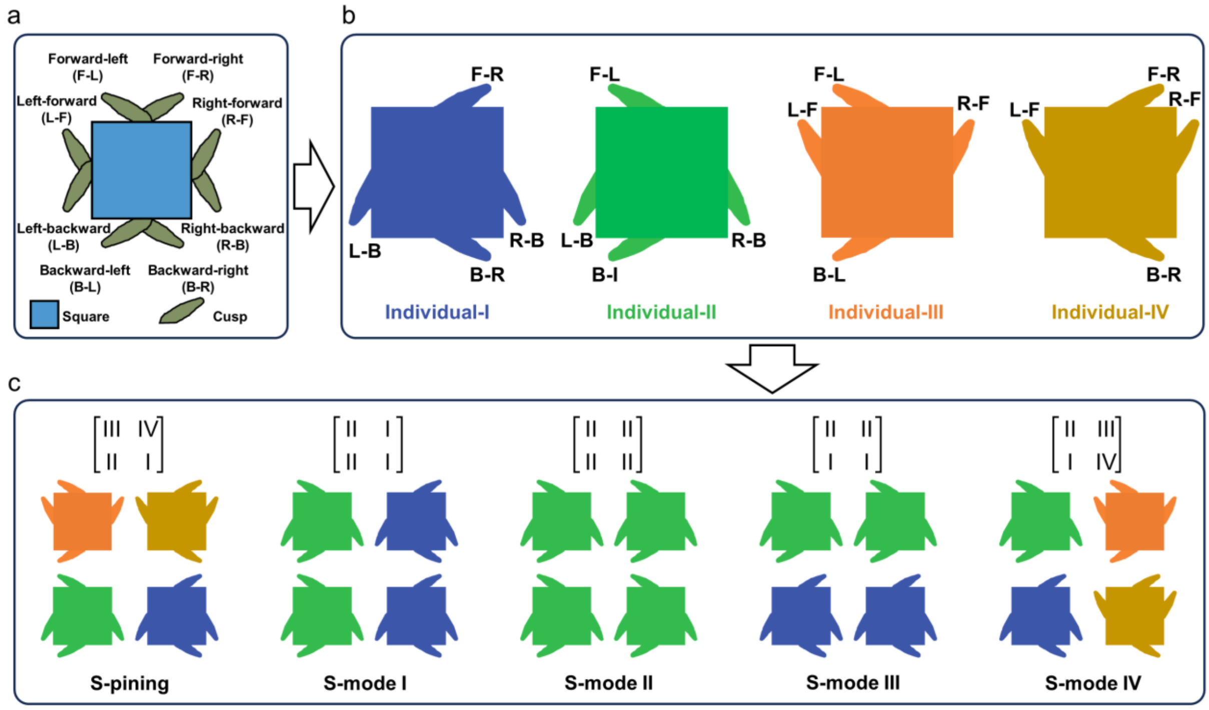

Fig. S4. Design of the square-cusp microstructure and its liquid spreading patterns. a Structural schematic of the square-cusp microstructure, featuring a square bulk with cusps arranged along its 4 edges. **b** Four distinct individual microstructure units (Individual I–IV) formed by varying cusp orientations, which determine the precursor film transport direction. **c** Five array patterns generated by systematically assembling the individual units, enabling precursor film pinning (S-pinning), as well as controlled unidirectional (S-mode I), bidirectional (S-mode II), tri-directional (S-mode III), and quad-directional (S-mode IV) spreading.

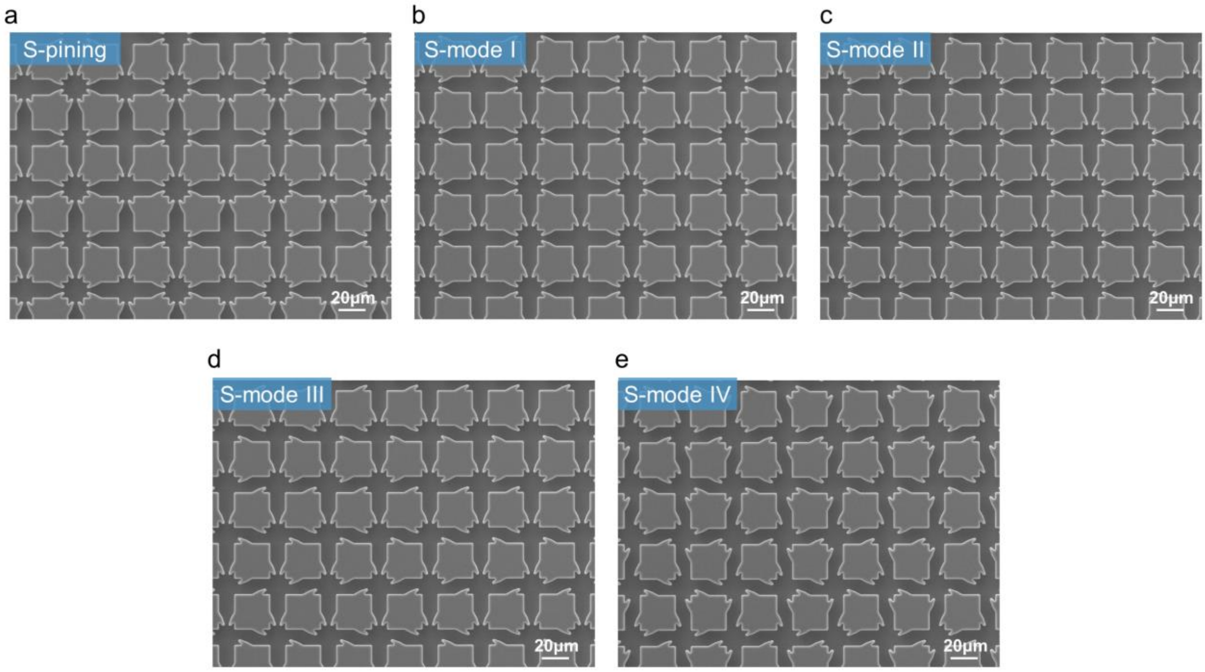

Fig. S5. SEM images of the square-cusp microstructures. a S-pinning mode, where the precursor film remains confined. **b** S-mode I, where the precursor film spreads in a single direction. **c** S-mode II, enabling bidirectional precursor film spreading along two directions. **d** S-mode III, where the precursor film expands in three directions. **e** S-mode IV, achieving quad-directional precursor film spreading.

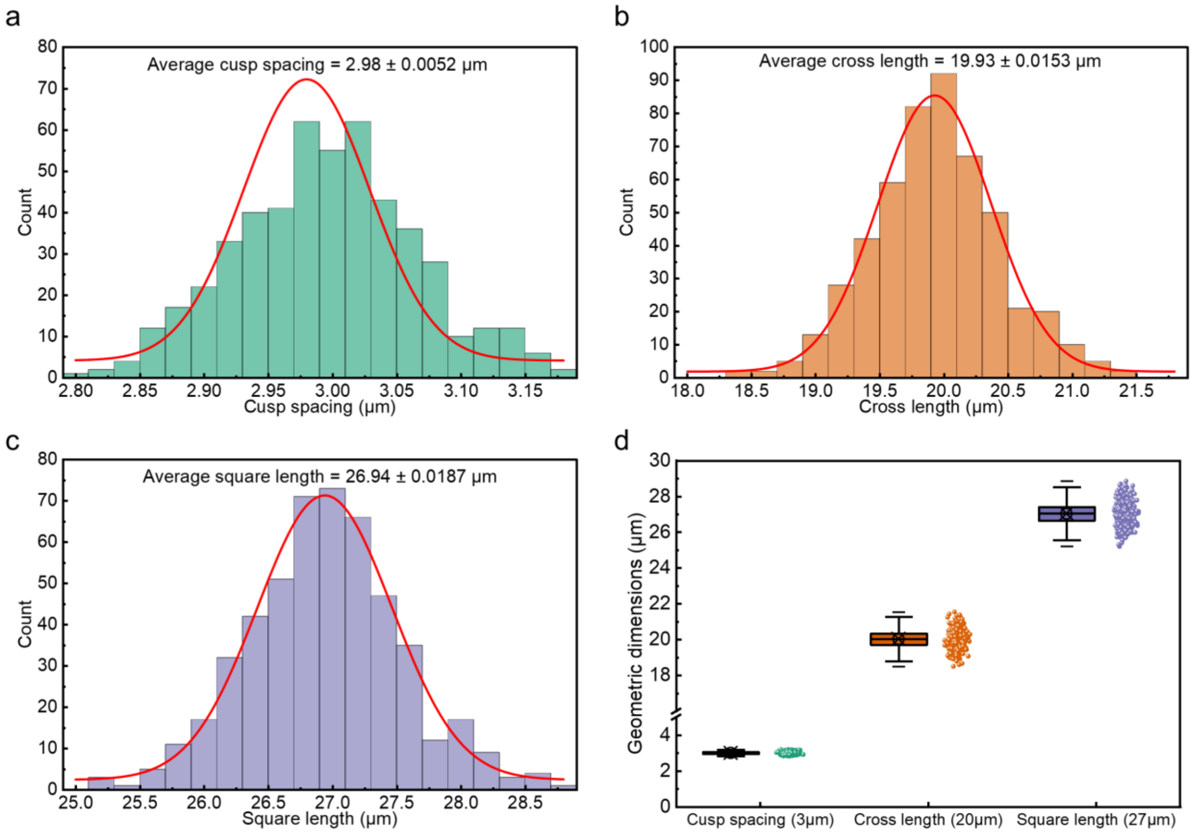

Fig. S6. Statistical analysis of feature size uniformity of the fabricated cross-cusp and square-cusp microstructures. a Histogram and Gaussian fitting of cusp spacing, based on 500 measured microstructures, showing an average value of 2.98 ± 0.0052 μm compared with the design value of 3 μm. **b** Histogram and Gaussian fitting of cross length, based on 500 measured microstructures, yielding an average of 19.93 ± 0.0153 μm compared with the design value of 20 μm. **c** Histogram and Gaussian fitting of square length, based on 500 measured microstructures, centered at 26.94 ± 0.0187 μm compared with the design value of 27 μm. **d** Scatter and box plots of cusp spacing, cross length, and square length, illustrating the dimensional statistics of the fabricated microstructures. Source data are provided as a Source Data file.

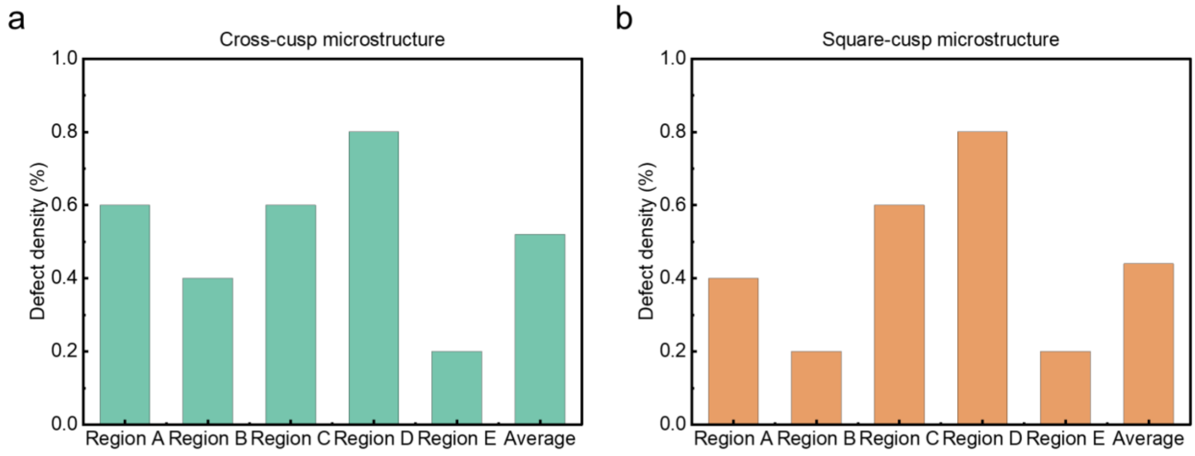

Fig. S7. Evaluation of defect density in the fabricated cross-cusp and square-cusp microstructures. a Defect density distribution of cross-cusp microstructures, calculated from 500 samples divided into 5 regions (A–E), with values ranging from 0.2% to 0.8% and an average defect density of about 0.52%. **b** Defect density distribution of square-cusp microstructures, calculated from 500 samples divided into 5 regions (A–E), with values ranging from 0.2% to 0.8% and an average defect density of about 0.44%. Source data are provided as a Source Data file.

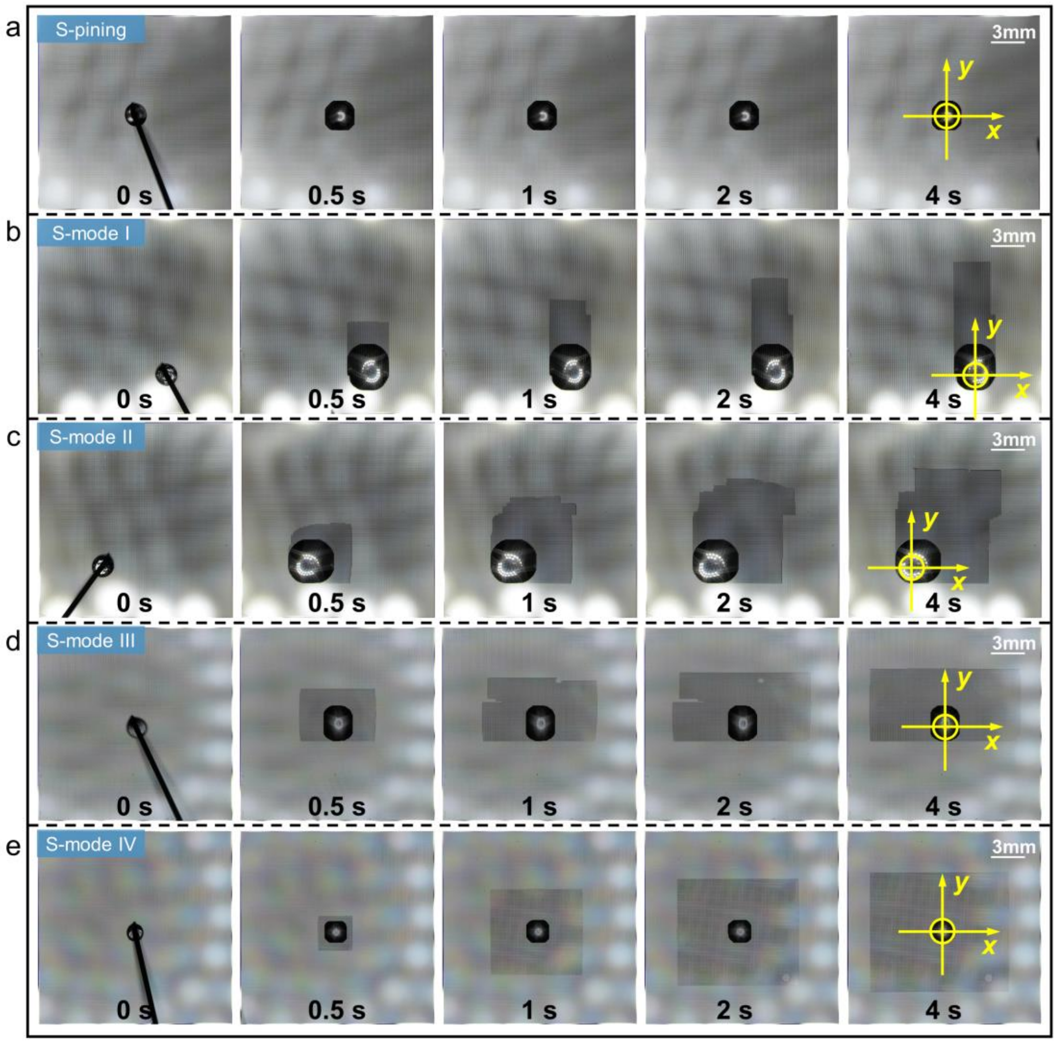

Fig. S8. Multi-directional liquid spreading behavior on the square-cusp microstructure. a-e Optical images of precursor film multi-directional spreading where the film remains pinned in S-pinning mode and spreads along 1 to 4 directions in S-mode I–IV, with no significant spreading of the droplet body.

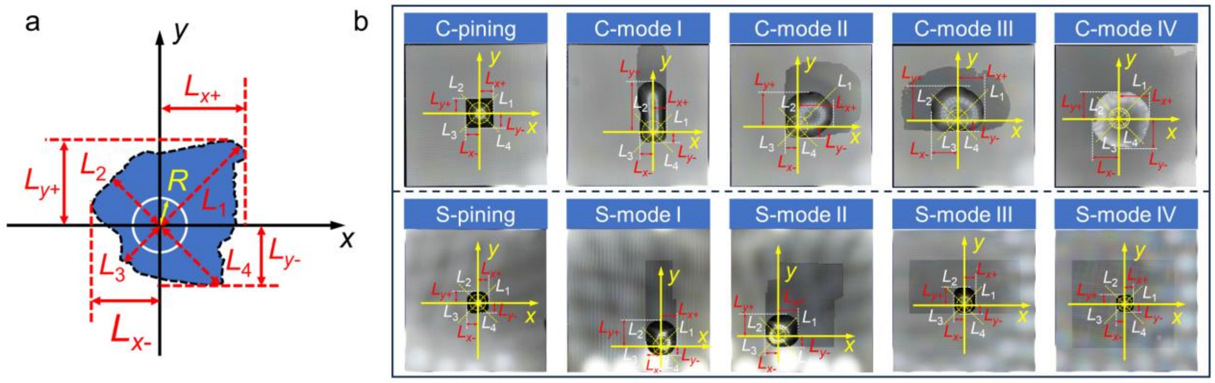

Fig. S9. Eight-directional normalized spreading-length analysis. a Schematic illustration of the measurement principle, where spreading lengths are defined along 4 Cartesian axes and 4 diagonal directions from the droplet center and normalized by the initial droplet radius R. **b** Representative examples showing the application of this method to different microstructured surfaces and spreading modes, with eight directional lengths labeled for clarity.

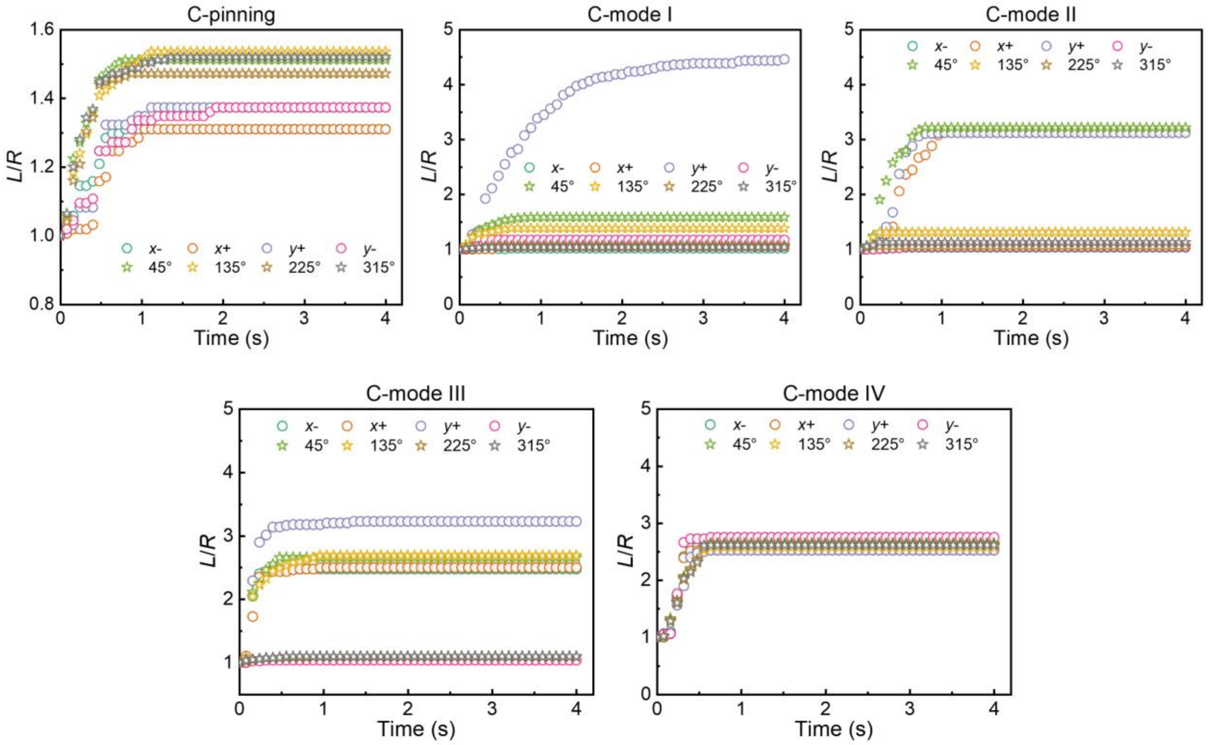

Fig. S10. Time-dependent normalized spreading lengths (*L*/*R*) of the droplet body along eight directions on cross-cusp microstructures in 5 spreading modes. All modes exhibit a rapid increase of L/R at the early stage (t < 1 s) followed by a plateau, indicating the establishment of stable spreading fronts. Distinct anisotropy is observed in guided modes, where one or several directions reach significantly larger normalized lengths compared to others, while isotropic cases maintain nearly overlapping curves across all directions. Source data are provided as a Source Data file.

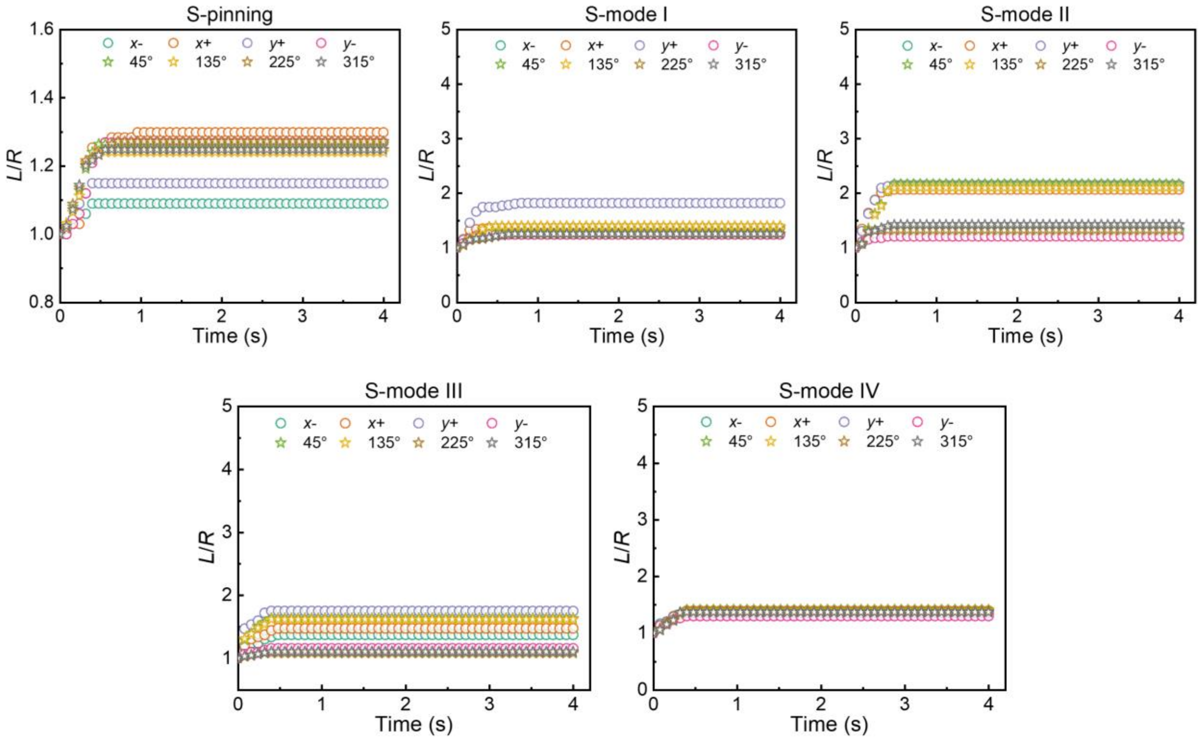

Fig. S11. Time-dependent normalized spreading lengths (L/R) of the droplet body along eight directions on square-cusp microstructures in 5 spreading modes. The droplet body here shows minimal extension, with L/R values remaining close to the initial radius. All modes exhibit nearly overlapping curves across the eight measured directions, indicating an overall isotropic spreading behavior with negligible directional preference. Source data are provided as a Source Data file.

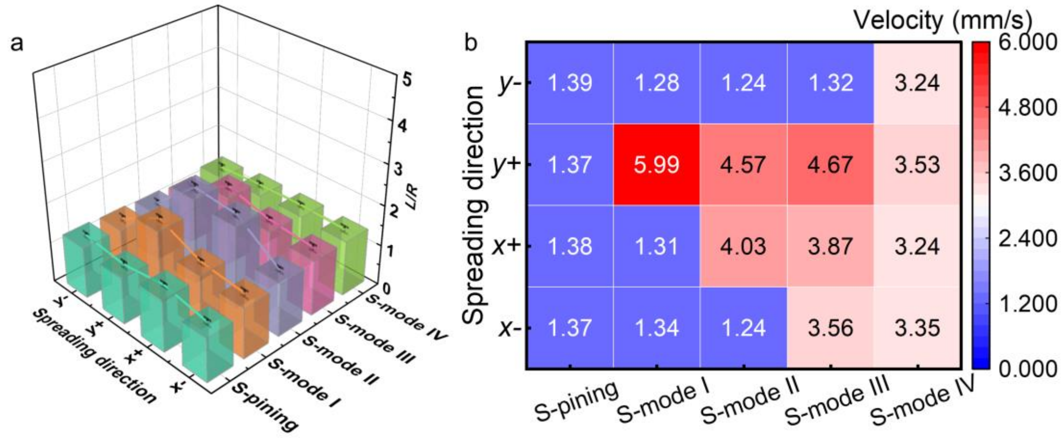

Fig. S12. Quantitative analysis of spreading distance and velocity on square-cusp microstructures in different modes. a Three-dimensional bar plots of normalized spreading lengths (L/R) in 4 directions, showing directional differences in spreading extent across 5 modes. Bars represent mean values, and error bars indicate mean ± SD (n =3 independent experiments). **b** Heatmap of maximum spreading velocity (mm/s) along each direction, highlighting the anisotropic dynamic behavior associated with square-cusp guided spreading. Source data are provided as a Source Data file.

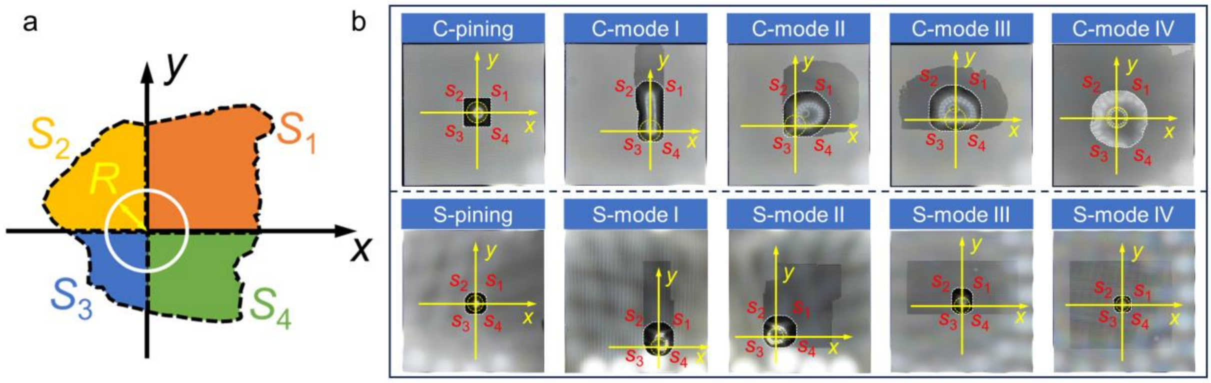

Fig. S13. Quadrant-based coverage area of droplets for global spreading quantification. a Schematic diagram showing the segmentation of the droplet-covered area into 4 quadrants (S_1 – S_4) centered at the droplet's geometric center, with the initial droplet radius R marked for normalization. **b** Representative steady-state spreading imprints on cross-cusp and square-cusp microstructures in different spreading modes, with quadrant areas labeled for quantitative comparison.

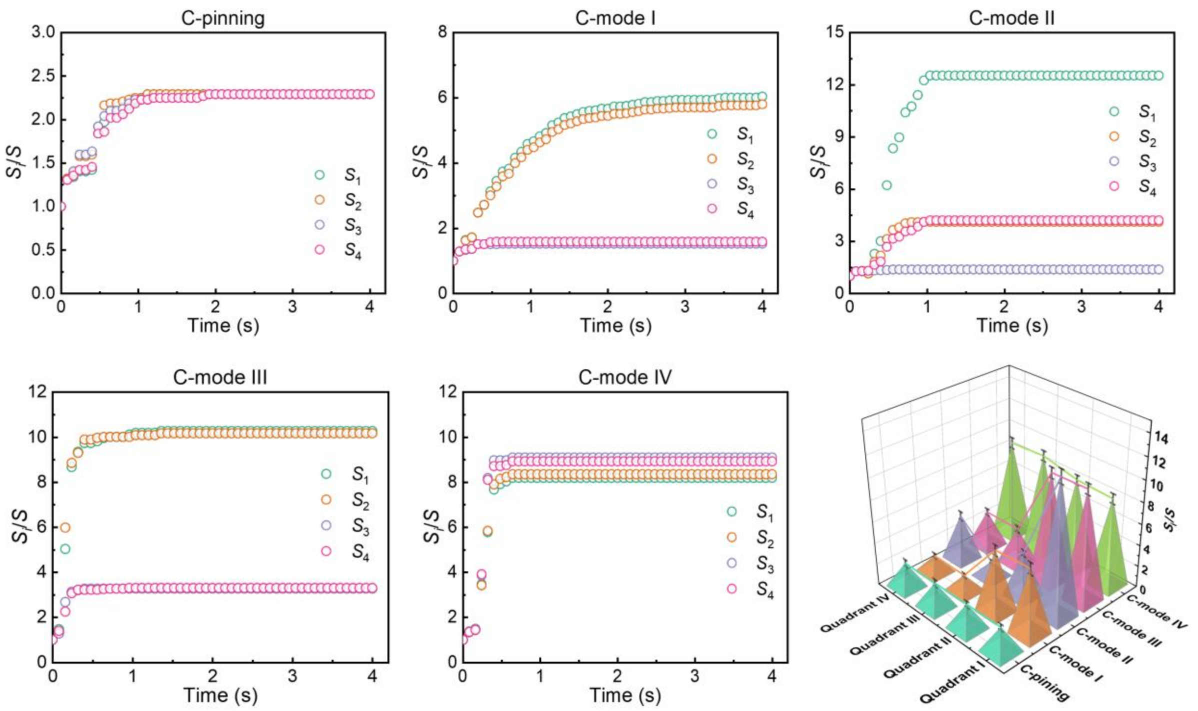

Fig. S14. Time-dependent normalized spreading areas (S_i/S) in 4 quadrants on cross-cusp microstructures for 5 spreading modes. The 3D summary plot further compares the contributions of each quadrant across all modes, highlighting the distinct differences in spreading anisotropy. C-pinning exhibits almost no spreading, with 4 quadrants maintaining nearly identical normalized areas. C-mode I shows strong elongation along the *y*-axis, leading to significantly larger spreading areas in the first and second quadrants. C-mode II is characterized by rapid spreading confined to the first quadrant, while other quadrants remain nearly unchanged. C-mode III demonstrates simultaneous spreading in both the first and second quadrants, producing highly anisotropic area growth. By contrast, C-mode IV displays relatively uniform expansion across all quadrants, indicative of nearly isotropic spreading. Bars represent mean values, and error bars indicate mean ± SD (*n* =3 independent experiments). Source data are provided as a Source Data file.

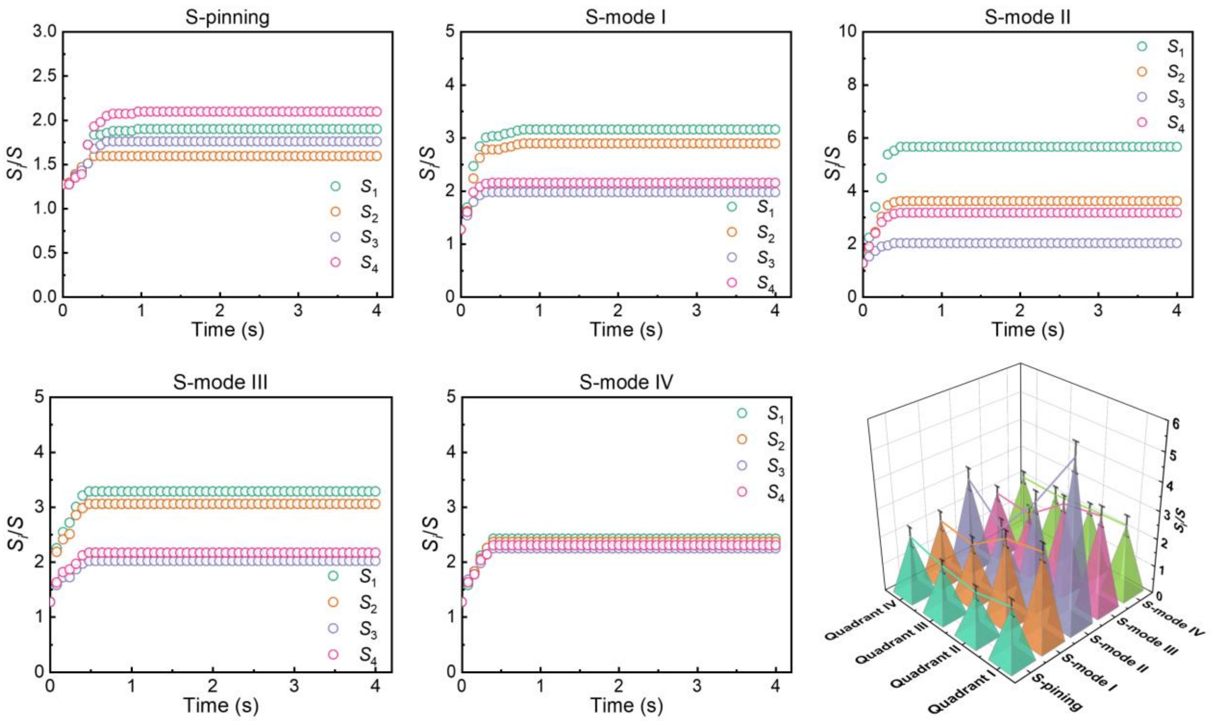

Fig. S15. Time-dependent normalized spreading areas (S_i/S) in 4 quadrants on square-cusp microstructures for 5 spreading modes. The 3D summary plot provides a comparison of quadrant contributions in all modes, revealing that overall spreading is limited and largely isotropic. In general, the droplet body shows little expansion, and quadrant areas remain relatively balanced with only minor anisotropy. Specifically, S-pinning shows almost no spreading, with all quadrants maintaining nearly constant normalized areas. S-mode I presents a slight elongation along the *y*-axis, giving somewhat larger areas in the first and second quadrants. S-mode II exhibits modest growth mainly in the first quadrant, while the other quadrants remain nearly unchanged. S-mode III shows limited expansion distributed between the first and second quadrants, but the extent remains small. S-mode IV displays nearly uniform spreading across all quadrants, reflecting an overall isotropic behavior. Bars represent mean values, and error bars indicate mean ± SD (n =3 independent experiments). Source data are provided as a Source Data file.

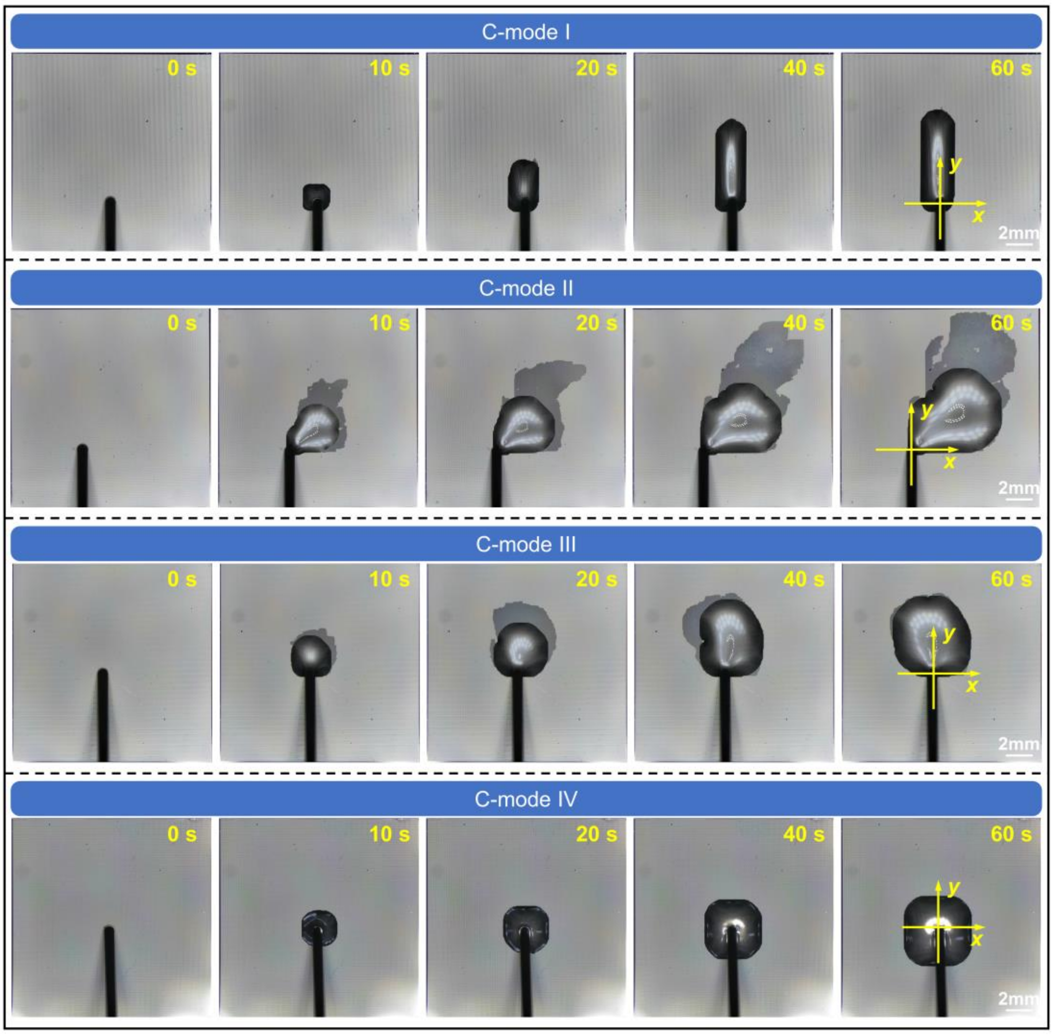

Fig. S16. Droplet spreading on cross-cusp surfaces under continuous injection in 4 representative modes (C-mode I–IV). Time-lapse images (0–60 s) show distinct spreading pathways. In C-mode I, the droplet spreads predominantly along the *y*+ direction; in C-mode II, spreading occurs simultaneously along the *x*+ and *y*+ directions; in C-mode III, spreading extends along the *x*+, *x*–, and *y*+ directions; while in C-mode IV, the droplet spreads uniformly along *x*+, *x*–, *y*+, and *y*– directions.

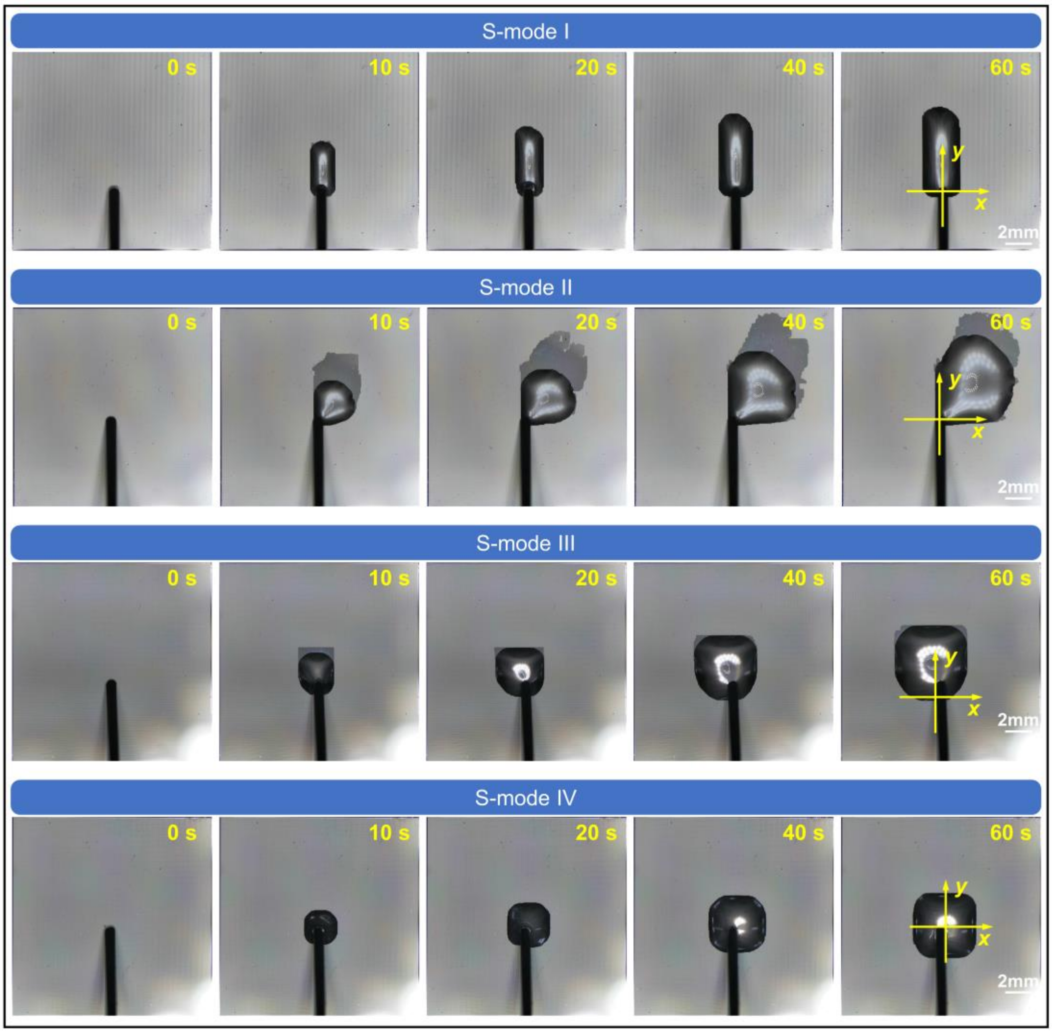

Fig. S17. Droplet spreading on square-cusp surfaces under continuous injection in 4 representative modes (S-mode I–IV). Time-lapse images (0–60 s) show distinct spreading pathways. In S-mode I, the droplet spreads predominantly along the $y+$ direction; in S-mode II, spreading occurs simultaneously along the $x+$ and $y+$ directions; in S-mode III, spreading extends along the $x+$, $x-$, and $y+$ directions; while in S-mode IV, the droplet spreads uniformly along $x+$, $x-$, $y+$, and $y-$ directions.

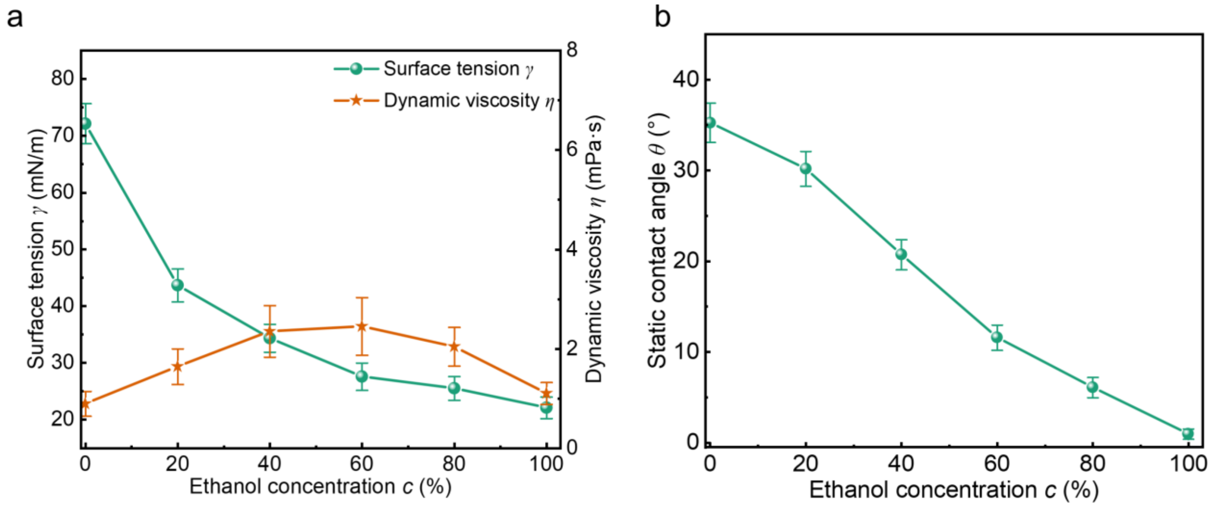

Fig. S18. Variation of ethanol–water mixture properties and wettability with ethanol concentration *c*. a Surface tension and dynamic viscosity of ethanol–water mixtures as a function of ethanol concentration, with surface tension decreasing sharply from about 72 mN/m at 0% ethanol to about 22 mN/m at 100% ethanol, while viscosity remains within 1–3 mPa·s across all concentrations. Bars represent mean values, and error bars indicate mean ± SD (n =3 independent experiments). **b** Static contact angle of ethanol–water mixtures on plasma-treated microstructured surfaces, decreasing monotonically from about 35° at 0% ethanol to nearly 0° at 100% ethanol. Bars represent mean values, and error bars indicate mean ± SD (n =3 independent experiments). Source data are provided as a Source Data file.

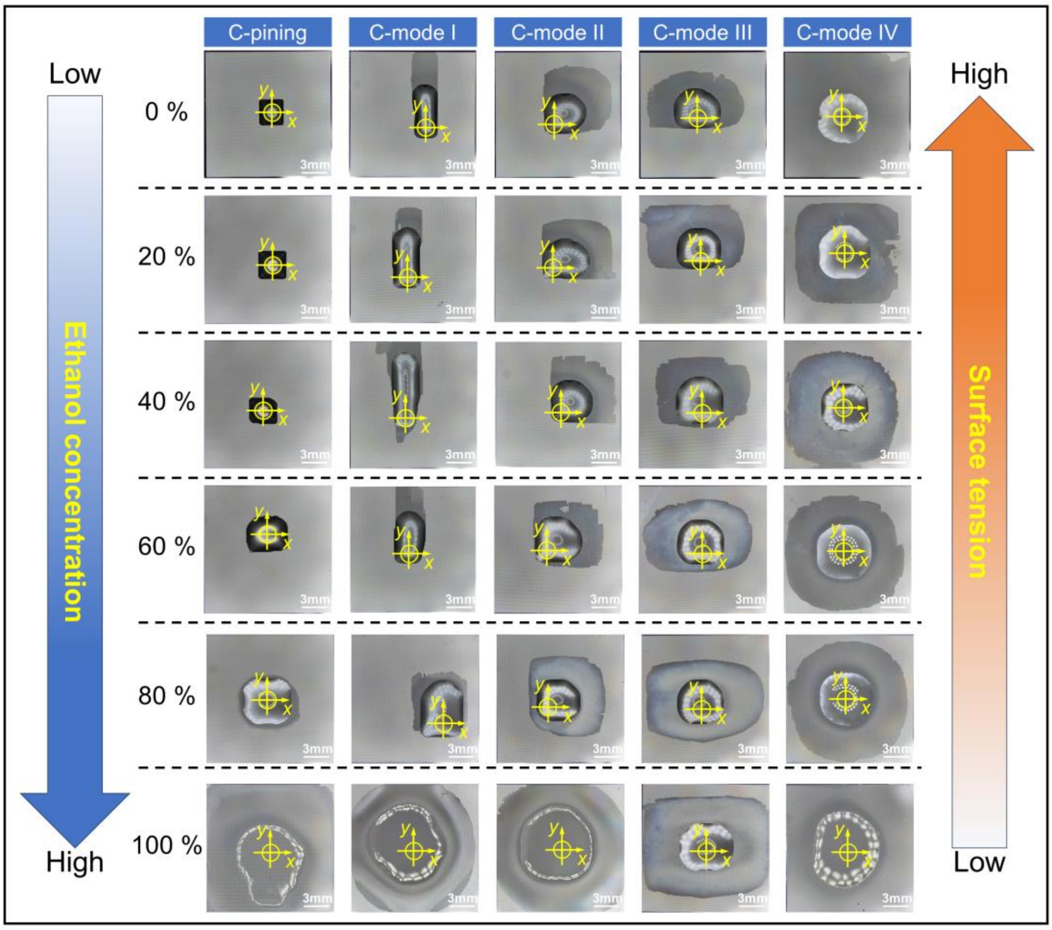

Fig. S19. Spreading behaviors of ethanol–water mixtures with different concentrations *c* on plasma-treated microstructured surfaces in 5 spreading modes (C-pinning, C-mode I–IV). As ethanol concentration increases from 0% to 100% and surface tension decreases, the spreading pattern evolves systematically: at low concentration (high surface tension), droplets mainly extend along the preset guided directions of each mode; at high concentration (low surface tension), enhanced wettability weakens pinning and triggers extension in nominally pinned directions, thereby reducing anisotropy.

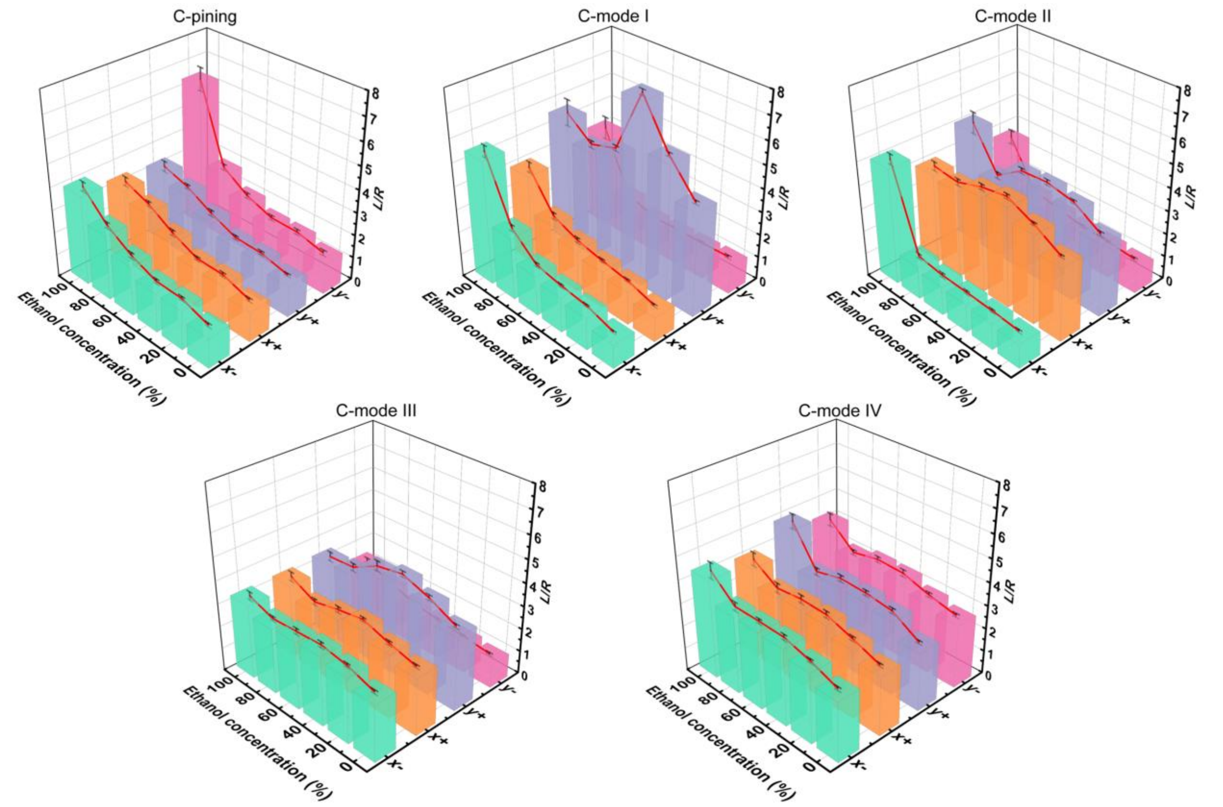

Fig. S20. Dimensionless spreading lengths of droplets with different ethanol concentrations *c* along *x*-, *x*+, *y*- and *y*+ directions in 5 spreading modes (C-pinning, C-mode I–IV). With increasing ethanol concentration, guided directions generally exhibit pronounced elongation at low concentrations ($c \leqslant 60\%$), followed by a decline at higher concentrations as directional confinement weakens, while pinned directions remain suppressed until surface tension is sufficiently reduced to induce leakage. In C-pinning, all 4 directions stay nearly uniform with gradual growth up to about 3 at 80%, then lose pinning completely in pure ethanol. In guided modes (C-mode I–III), the preferred directions peak around 40–60% ethanol before decreasing, whereas in C-mode IV, all 4 axes increase progressively, reflecting a transition from anisotropic to nearly isotropic spreading at high ethanol contents. Bars represent mean values, and error bars indicate mean ± SD (*n* =3 independent experiments). Source data are provided as a Source Data file.

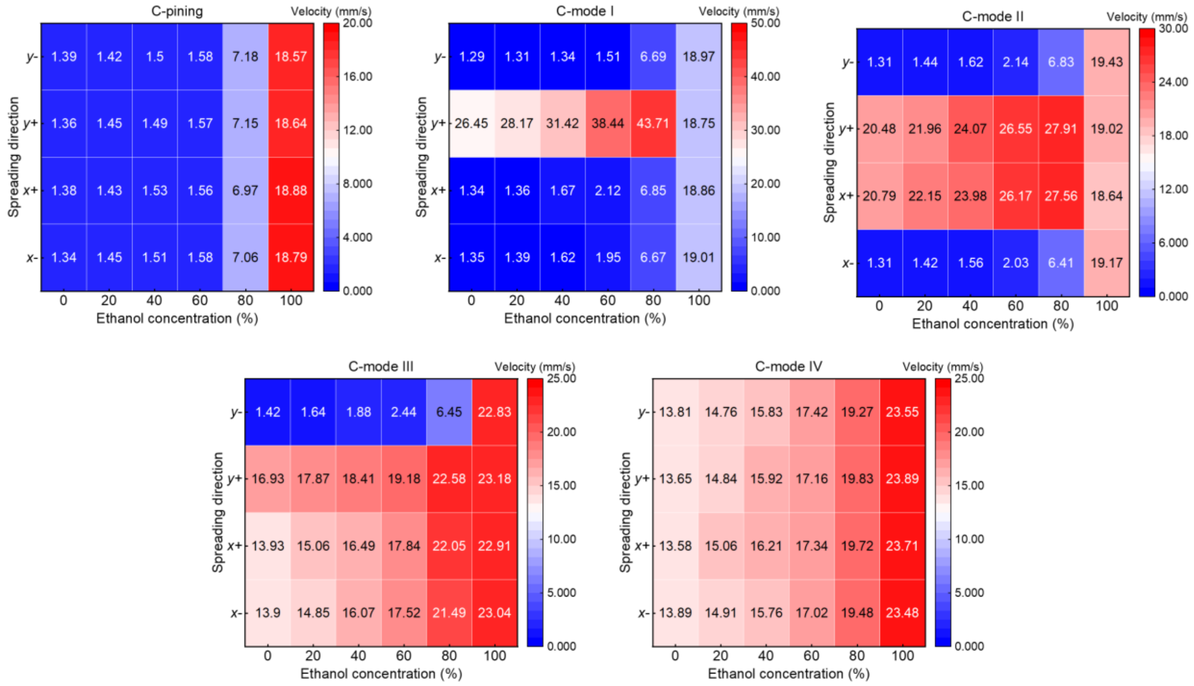

Fig. S21. Spreading velocities of droplets with different ethanol concentrations c along x-, x+, y- and y+ directions in 5 spreading modes (C-pinning, C-mode I–IV). The results show a general trend in which guided directions accelerate steadily with increasing ethanol concentration and reach maxima near 80%, while pinned directions remain very slow (<1.5 mm·s^{-1}) until high concentrations reduce pinning. At pure ethanol, enhanced wettability removes anisotropy, producing high velocities in all directions, though slightly below the peak values at 80%. In C-pinning, velocities are uniformly low at $c \leqslant 60\%$ ethanol, then rise sharply at 80–100% as pinning weakens. In guided modes (C-mode I–III), the desired directions (e.g., y+ in C-mode I) exhibit sharp acceleration up to about 40–43 mm·s^{-1} at 80%, whereas pinned axes remain near baseline. In C-mode IV, all 4 directions increase progressively with concentration, indicating isotropic acceleration. Overall, mixtures with $c \leqslant 60\%$ ethanol sustain anisotropic spreading dominated by guided axes, while $c \geqslant 80\%$ ethanol leads to nearly uniform spreading behavior. Source data are provided as a Source Data file.

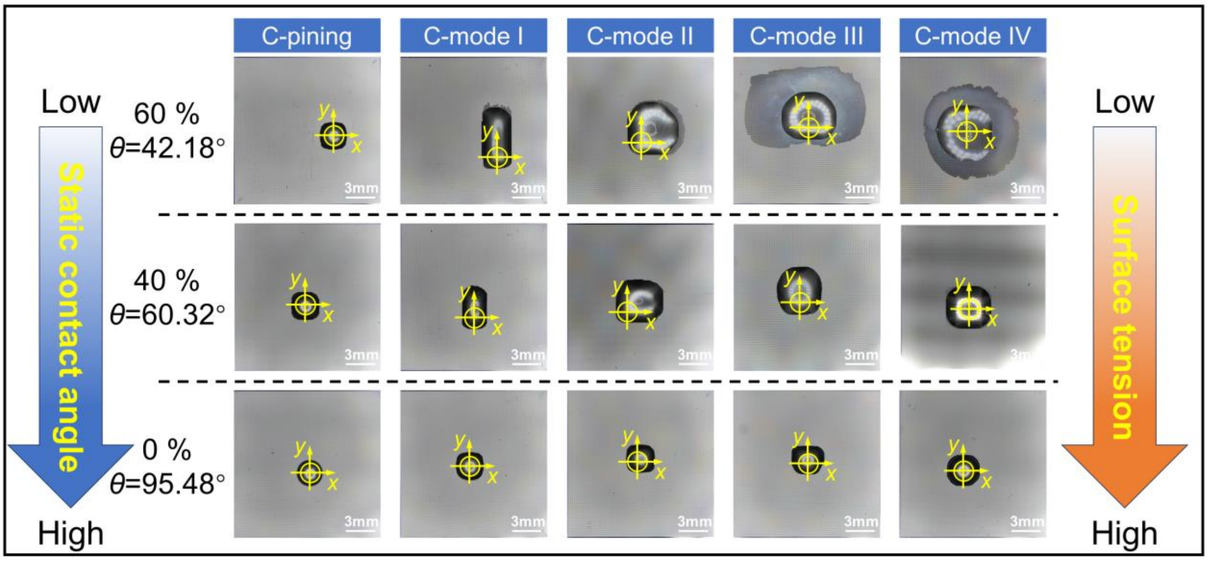

Fig. S22. Spreading behaviors of ethanol–water mixtures with different concentrations *c* on plasma-treated microstructured surfaces in 5 spreading modes (C-pinning, C-mode I–IV). The results show that at small contact angles (e.g., 60% ethanol, CA=42.2°), droplets spread readily along multiple desired directions with long distances and strong anisotropy. At intermediate wettability (40% ethanol, CA=60.3°), spreading remains multi-directional but is reduced in range, with precursor films becoming less continuous along guided axes. At high contact angle (pure water, CA=95.5°), spreading is strongly suppressed, as both droplet bodies and precursor films lose directionality and remain pinned regardless of the spreading mode.

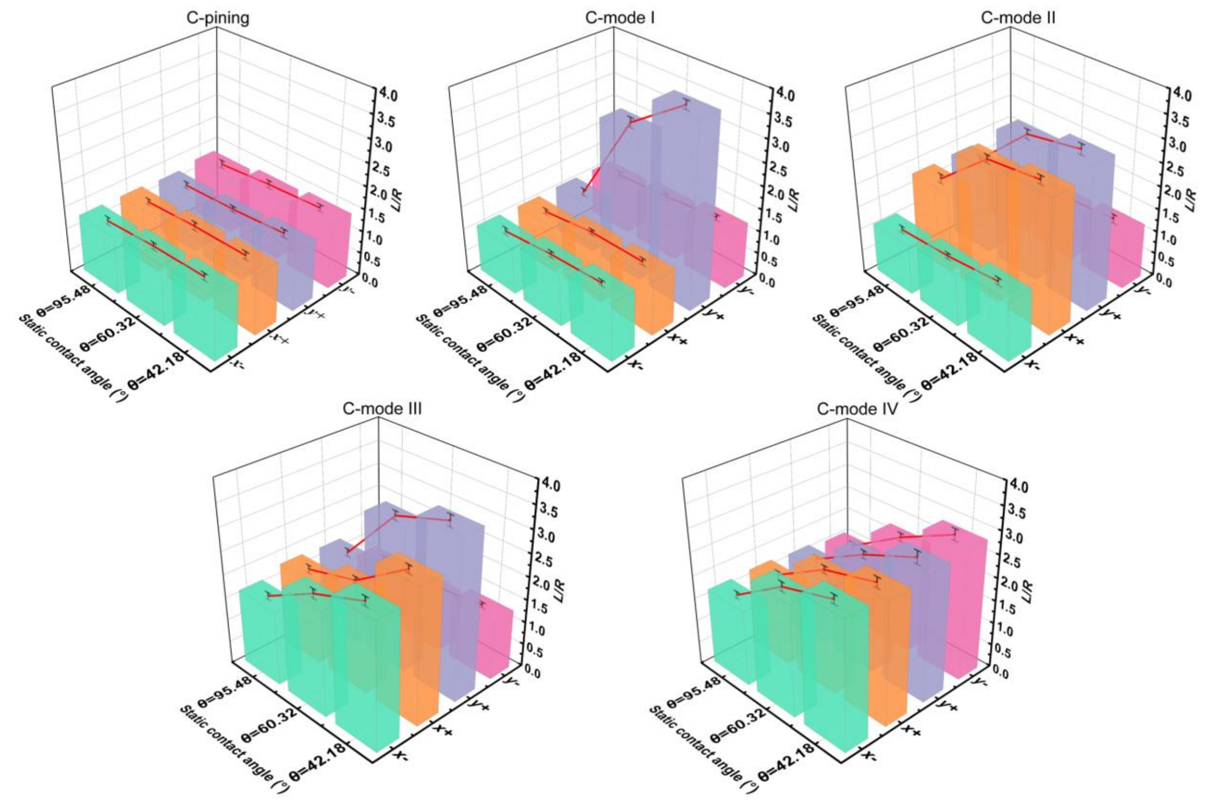

Fig. S23. Dimensionless spreading lengths of droplets with different ethanol concentrations *c* along *x*-, *x*+, *y*- and *y*+ directions in 5 spreading modes (C-pinning, C-mode I–IV). Liquids with low contact angles, such as 60% ethanol (CA=42.2°), exhibit the longest spreading lengths along desired directions, typically reaching more than two to three times the initial radius, while pinned directions remain near unity. At intermediate contact angles (about 60.3°), the spreading lengths decrease moderately, reflecting weakened directional guidance. For pure water with the high contact angle (CA=95.5°), spreading is strongly suppressed, with all directions showing values close to one regardless of the spreading mode, indicating the near-complete loss of anisotropy. Bars represent mean values, and error bars indicate mean ± SD (*n* =3 independent experiments). Source data are provided as a Source Data file.

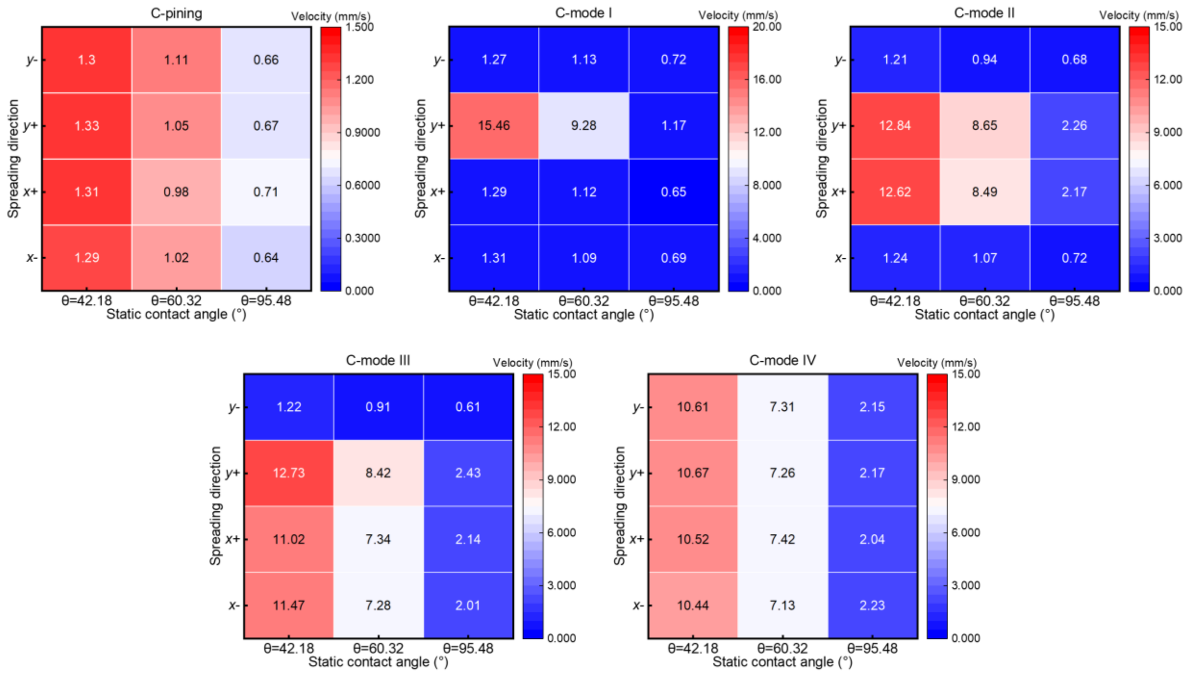

Fig. S24. Spreading velocities of droplets with different ethanol concentrations *c* along *x*-, *x*+, *y*- and *y*+ directions in 5 spreading modes (C-pinning, C-mode I–IV). Surfaces with low contact angles, such as 60% ethanol (CA=42.2°), achieve rapid spreading along guided directions, often exceeding 10 mm·s^{-1}, while pinned directions remain below 2 mm·s^{-1}. At intermediate contact angles (about 60.3°), the velocities decrease accordingly, reflecting weakened guidance. When the static contact angle is high (CA=95.5°, pure water), velocities remain uniformly low across all directions, confirming that increased hydrophobicity suppresses precursor film spreading and eliminates anisotropic extension. These results highlight that static contact angle governs not only the extent but also the velocity of directional spreading, with low angles favoring efficient guided spreading and high angles resulting in pinned states. Source data are provided as a Source Data file.

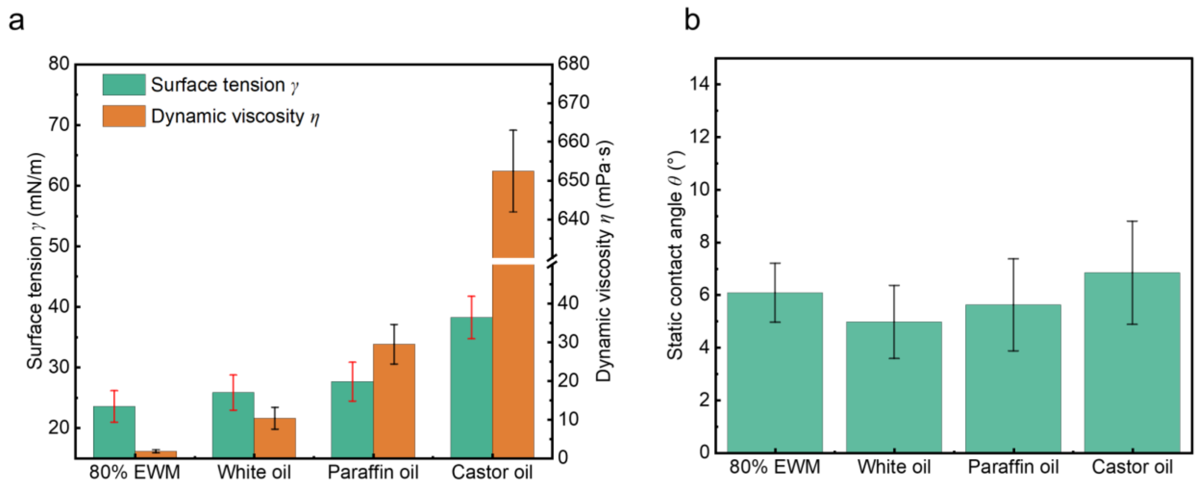

Fig. S25. Surface tension, dynamic viscosity and static contact angle of 4 different liquids (80% ethanol–DI water mixtures (80% EWM), white oil, paraffin oil, and castor oil). a Surface tension and dynamic viscosity of the 4 kinds of liquids, showing that although their surface tensions are comparable in the range of 25–40 $mN \cdot m^{-1}$, their viscosities differ significantly, from below 10 mPa·s for ethanol solution and white oil to several hundred mPa·s for castor oil. Bars represent mean values, and error bars indicate mean ± SD (*n* =3 independent experiments). **b** Static contact angles of all 4 kinds of liquids on plasma-treated microstructured surfaces remain below 10°, confirming similar wetting conditions. Bars represent mean values, and error bars indicate mean ± SD (*n* =3 independent experiments). Source data are provided as a Source Data file.

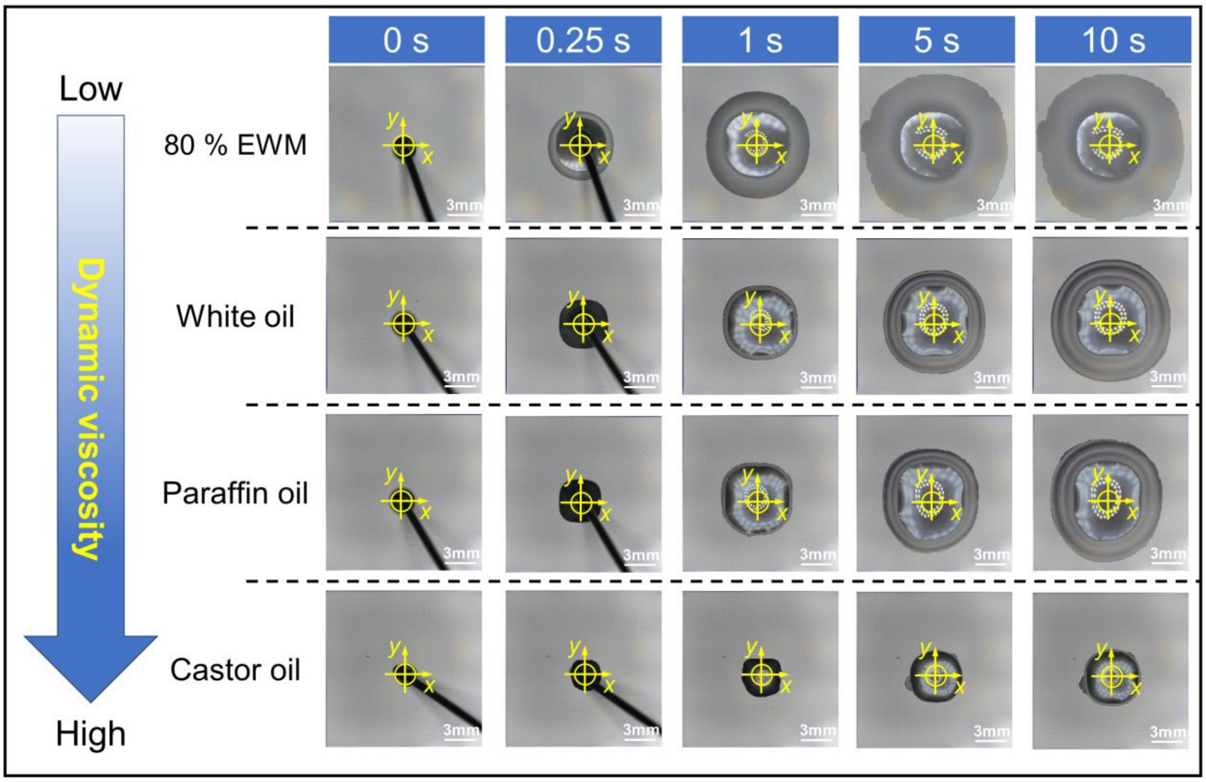

Fig. S26. Time-sequence images of droplet spreading in the C-mode IV microstructured surface for 80% ethanol–DI water mixtures (EWM), white oil, paraffin oil, and castor oil. For the low-viscosity liquid (c≥80% EWM), spreading is rapid and reaches equilibrium within a few seconds, with smooth and extensive precursor film propagation. For liquids with intermediate viscosity (white oil and paraffin oil), spreading slows down and the final coverage decreases, though isotropic patterns are maintained in this mode. For the highest-viscosity liquid (castor oil), spreading is strongly suppressed, and the droplet remains nearly pinned even after 10 s. These results confirm that while surface tension provides the driving force, viscosity governs the rate and extent of multi-directional spreading.

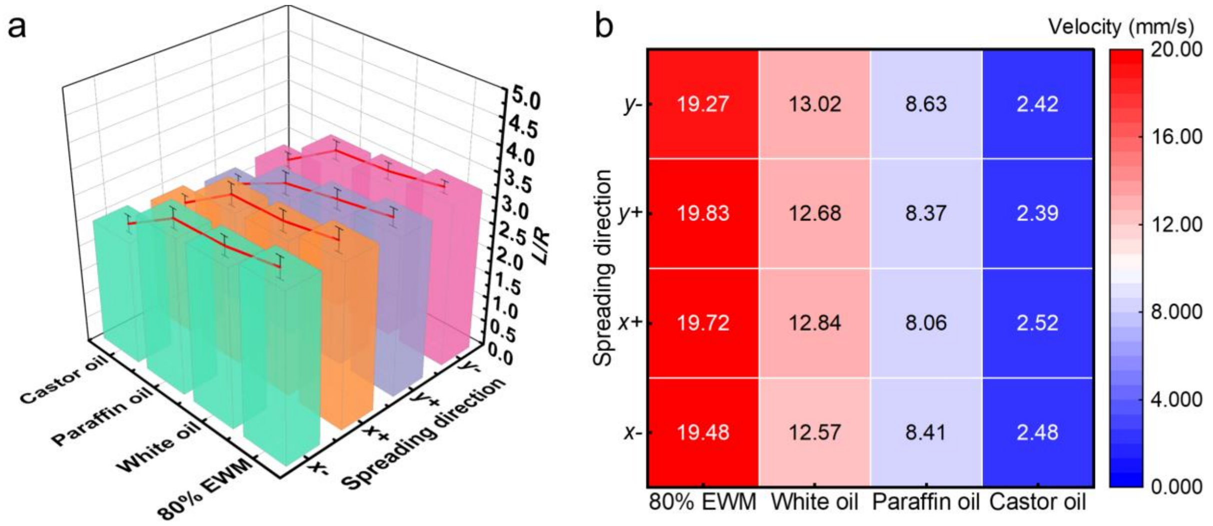

Fig. S27. Quantitative analysis of multi-directional spreading for 80% ethanol–DI water mixtures (EWM), white oil, paraffin oil, and castor oil with different viscosities. a Dimensionless spreading length of the 4 liquids, showing that viscosity has only a modest effect on spreading length: ethanol, white oil, and paraffin oil all maintain values above 2 along desired directions, while only castor oil with the highest viscosity (about 650 mPa·s) exhibits a markedly reduced value close to 2. Bars represent mean values, and error bars indicate mean ± SD (n =3 independent experiments). **b** Spreading velocities of the 4 kinds of liquids along x-, x+, y-, and y+ directions, demonstrating that viscosity strongly governs spreading speed: ethanol spreads fastest with peak velocities near 20 mm/s, white oil and paraffin oil show moderate reductions (12–13 mm/s and 8–9 mm/s, respectively), whereas castor oil spreads extremely slowly at about 2.5 mm/s. These results indicate that spreading length is largely maintained across low- and medium-viscosity liquids due to microstructure-guided transport, but spreading speed is decisively suppressed as viscosity increases. Source data are provided as a Source Data file.

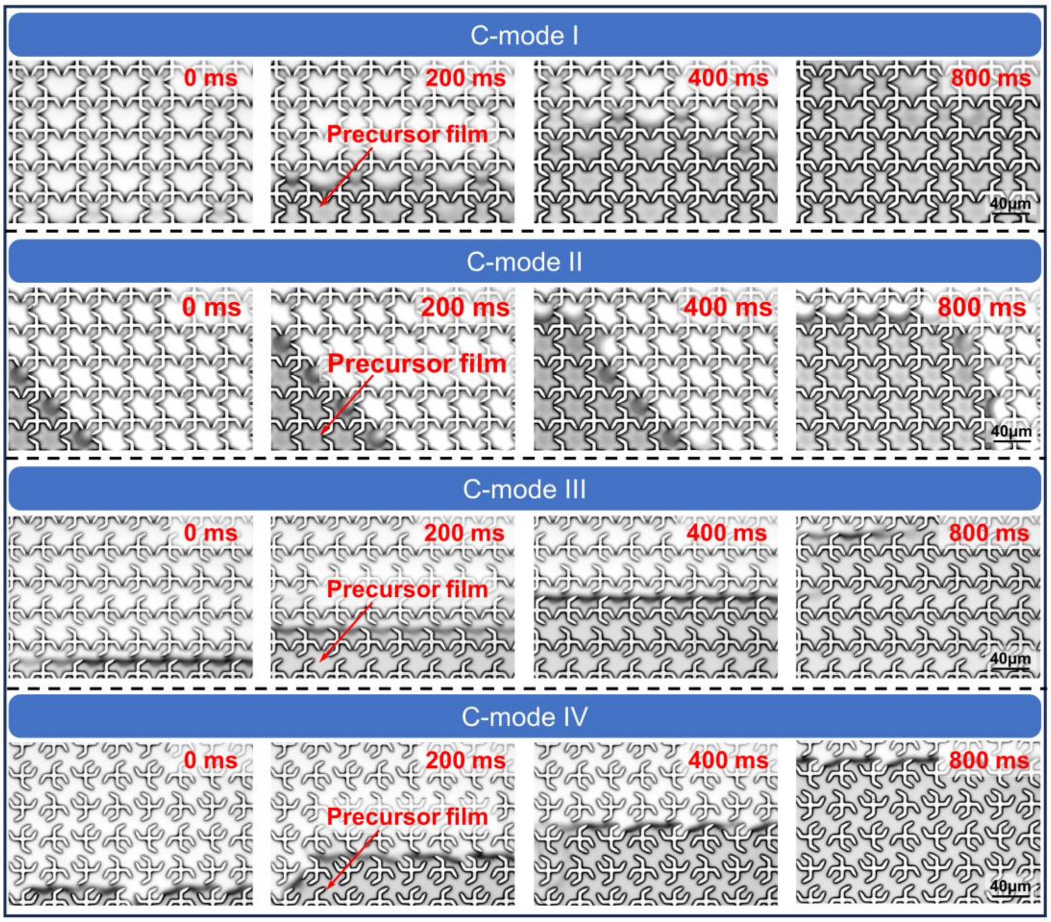

Fig. S28. Sequential images showing precursor film spreading on a cross-cusp structured surface in 4 spreading modes (C-mode I–IV). In all modes, the precursor film emerges rapidly within 200 ms and extends along the desired directions, forming continuous pathways across the microstructure. As time progresses (400–800 ms), the film further propagates to cover larger areas, demonstrating ultrafast wetting dynamics and strong anisotropic guidance imposed by the cusp arrays.

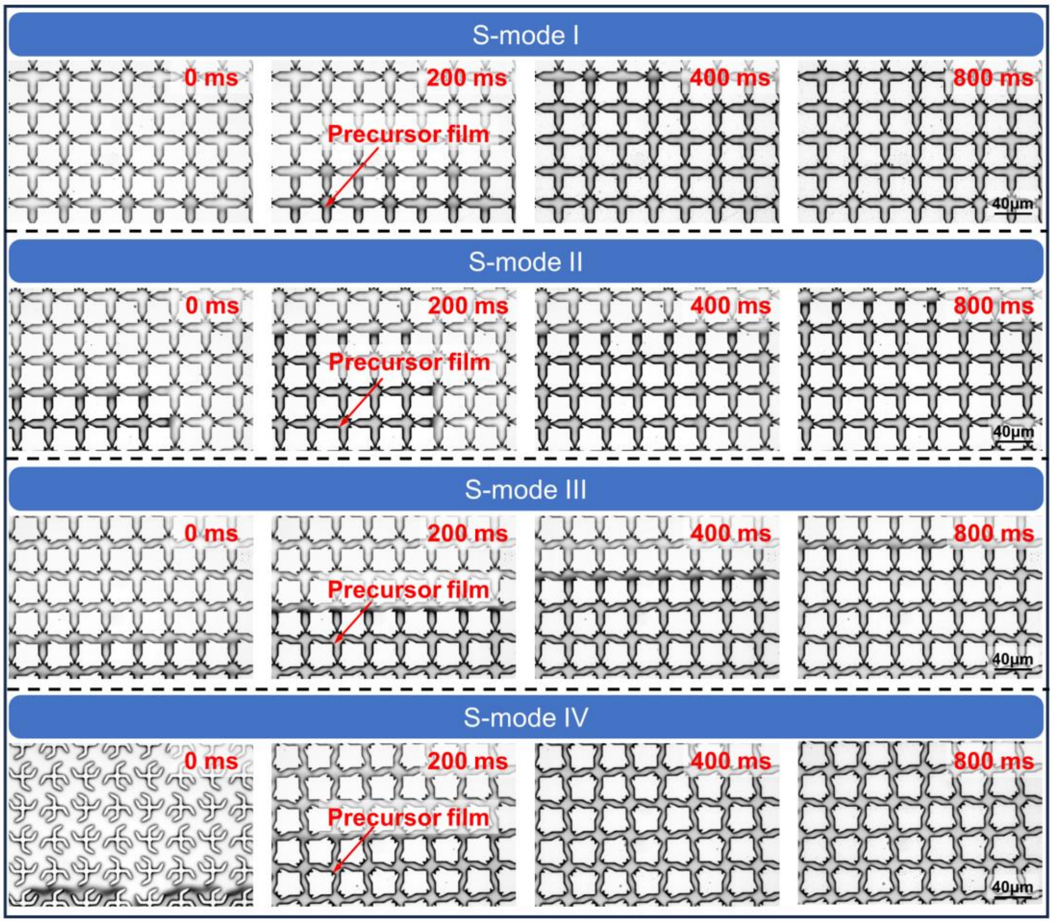

Fig. S29. Sequential images showing precursor film spreading on a square-cusp structured surface in 4 spreading modes (S-mode I–IV). In all modes, the precursor film emerges rapidly within 200 ms and extends along the desired directions, forming continuous pathways across the microstructure. As time progresses (400–800 ms), the film further propagates to cover larger areas, demonstrating ultrafast wetting dynamics and strong anisotropic guidance imposed by the cusp arrays.

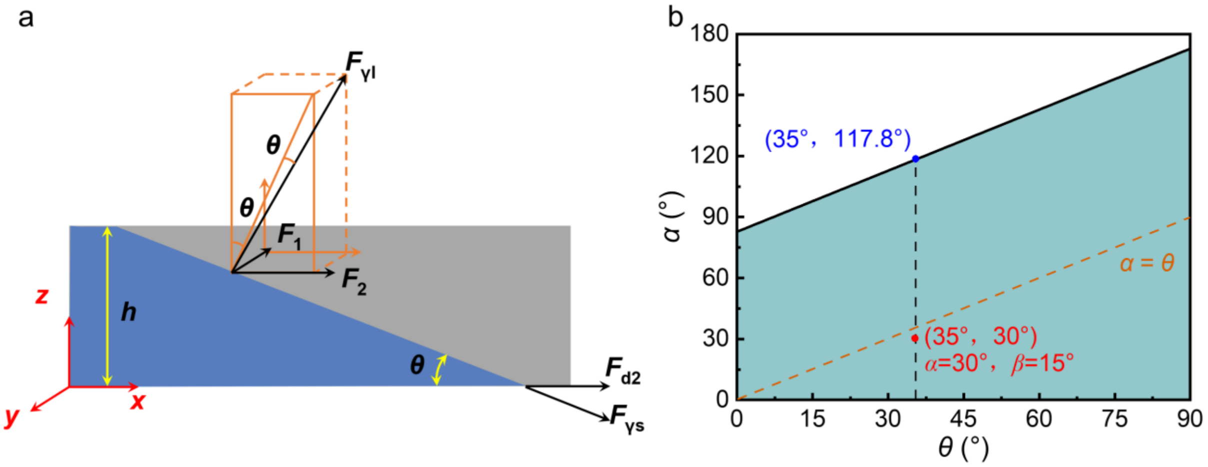

Fig. S30. Force analysis related to precursor film spreading and backward pinning. a Schematic of capillary force decomposition during precursor film spreading on the bulk-cusp microstructure. **b** Design space for backward pinning defined by the intrinsic contact angle θ and cusp-to-bulk angle α, where the shaded region satisfies the condition for effective precursor film pinning. Source data are provided as a Source Data file.

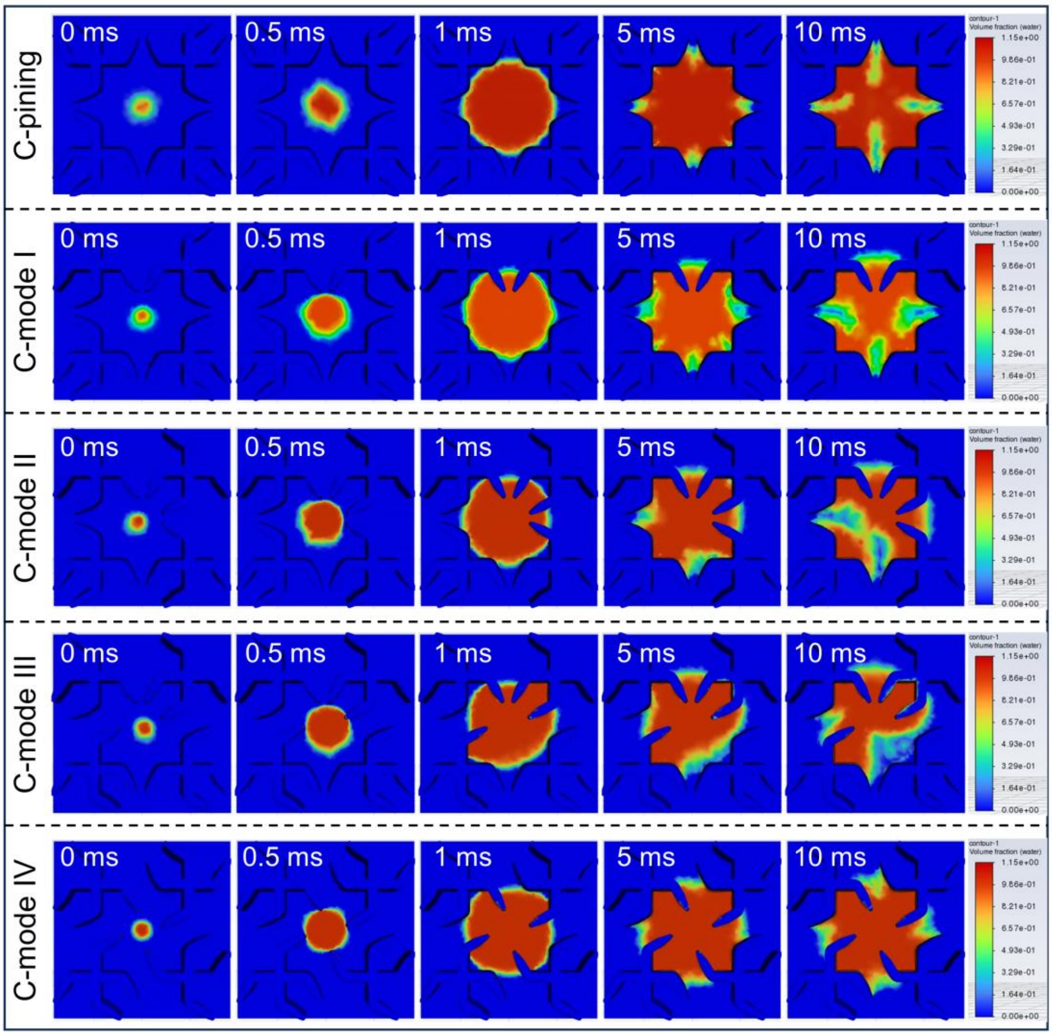

Fig. S31. CFD simulation of time-resolved droplet spreading on cross-cusp microstructures in 5 modes (C-pinning, C-mode I–IV). The color maps represent normalized liquid fraction intensity, with red indicating high values. Within the first 1 ms, droplets spread rapidly outward, forming precursor films that interact with the cusp structures. From 5 ms to 10 ms, distinct anisotropic spreading patterns emerge, where C-mode I shows preferential extension along the *y*+ axis, C-mode II and III exhibit directional spreading toward multiple axes, and C-mode IV displays nearly symmetric expansion in 4 directions, highlighting the guiding role of cusp geometry.

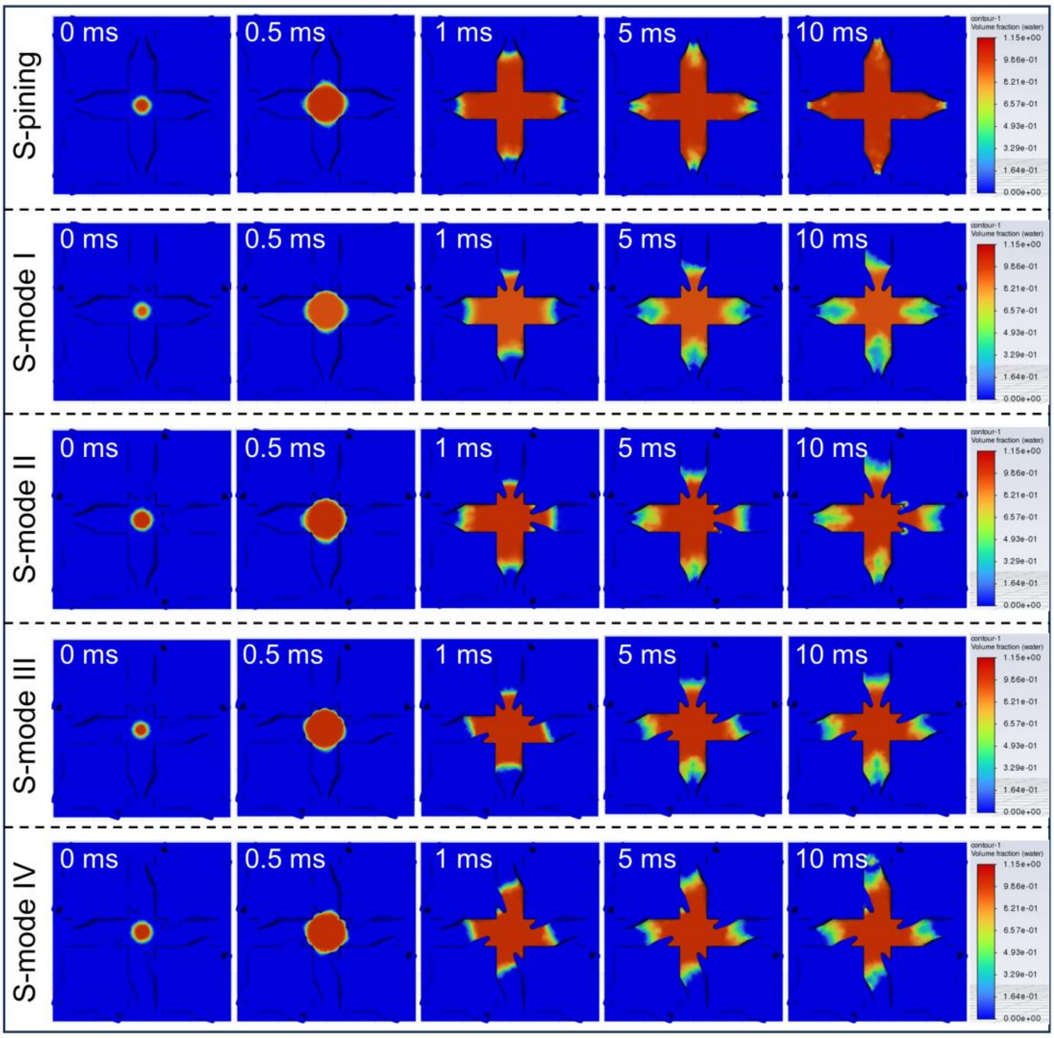

Fig. S32. CFD simulation of time-resolved droplet spreading on square-cusp microstructures in 5 modes (S-pinning, S-mode I–IV). The color maps represent normalized liquid fraction intensity, with red indicating high values. At 0–1 ms, droplets spread rapidly to form precursor films constrained by the square-cusp geometry. From 5 ms to 10 ms, the spreading patterns further evolve and stabilize, with each mode showing characteristic extensions along specific directions, reflecting the guiding role of the square-cusp microstructures.

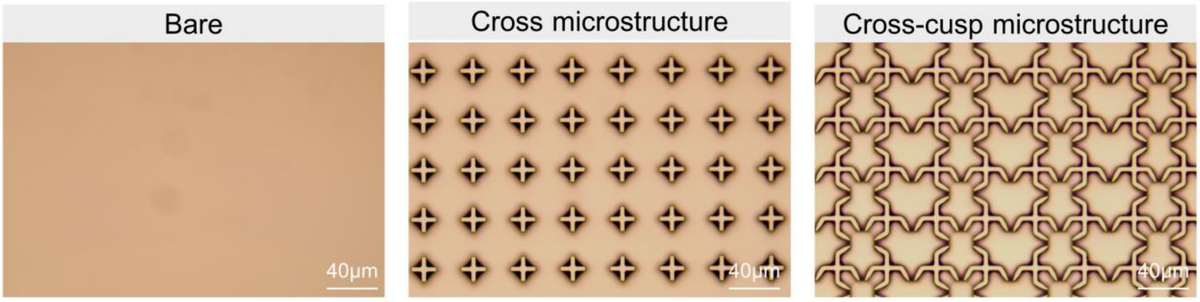

Fig. S33. Optical images of 3 types of surfaces including bare surface, cross microstructured surface, and cross-cusp microstructured surface. The bare surface shows a featureless flat morphology, while the cross microstructured surface consists of regularly arranged cross-shaped units. The cross-cusp microstructured surface further introduces cusp tips, forming interconnected paths that provide additional guidance and pinning effects for droplet spreading.

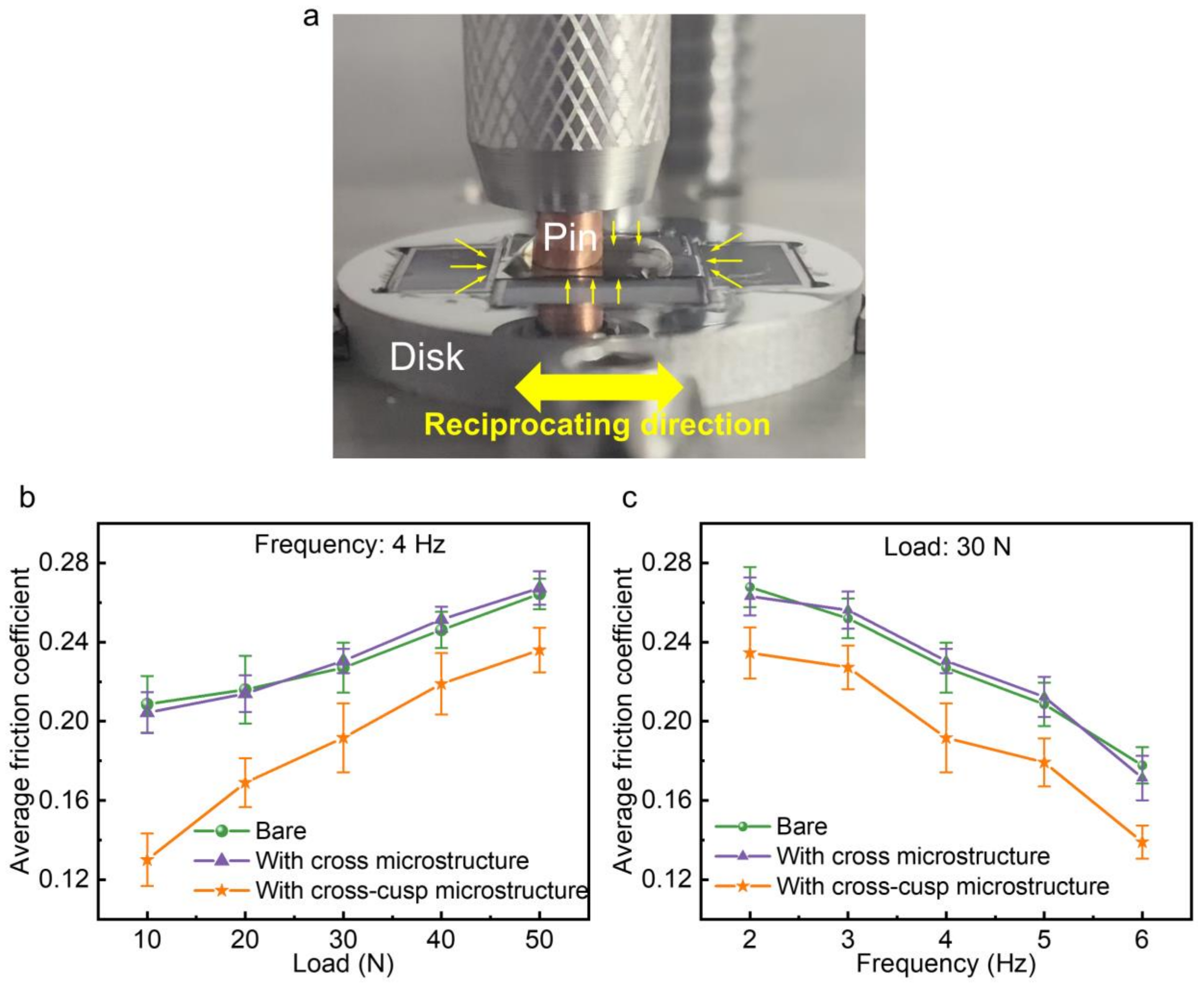

Fig. S34. Tribological performance evaluation of microstructured and bare surfaces under variable conditions. a Schematic of the pin-on-disk tribological test under water lubrication. **b** Average friction coefficients of bare surface, cross microstructured surface and cross-cusp microstructured surface measured at different normal loads (10–50 N) under a fixed frequency of 4 Hz. Bars represent mean values, and error bars indicate mean ± SD (*n* =3 independent experiments). **c** Average friction coefficients under different sliding frequencies (2–6 Hz) at a fixed load of 30 N. In both cases, the cross-cusp micro-structured surface exhibits consistently lower friction, confirming enhanced lubrication across varying mechanical conditions. Bars represent mean values, and error bars indicate mean ± SD (*n* =3 independent experiments). Source data are provided as a Source Data file.

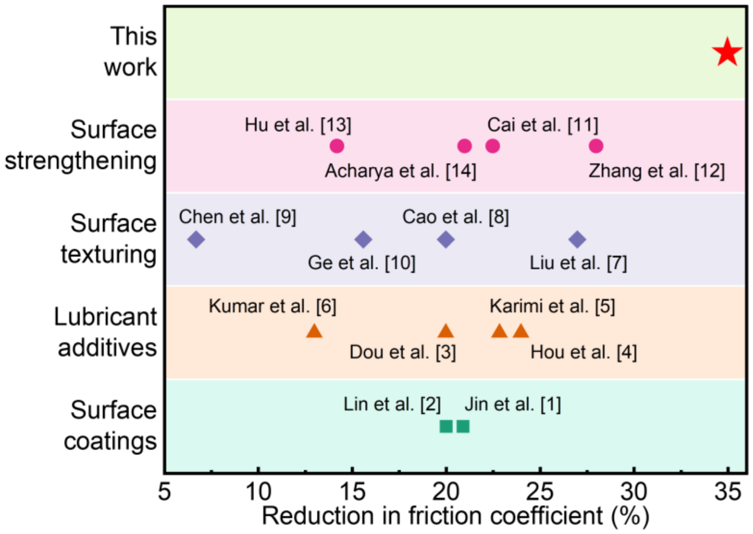

Fig. S35. Comparison of friction coefficient reduction achieved by this work and representative advanced lubrication strategies. The results are categorized into 4 conventional approaches—surface coatings, lubricant additives, surface texturing, and surface strengthening—each showing a typical reduction of 10–25%. In contrast, the present work (red star) achieves a markedly higher reduction of about 35%, highlighting the superior performance of the proposed microstructured strategy compared with existing state-of-the-art methods.

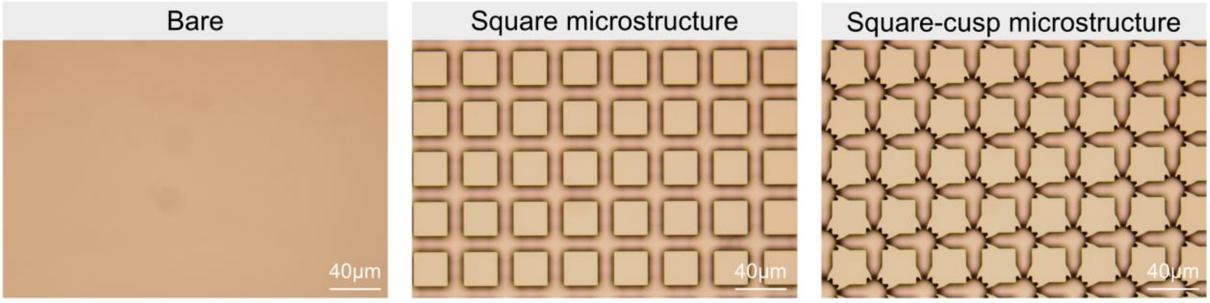

Fig. S36. Optical images of bare surface, square microstructured surface, and square-cusp microstructured surface. The bare surface shows a flat morphology without microfeatures, the square microstructured surface is composed of regularly arranged square units, and the square-cusp microstructured surface integrates cusps, forming interlinked edges that enhance directional guidance and pinning during droplet spreading.

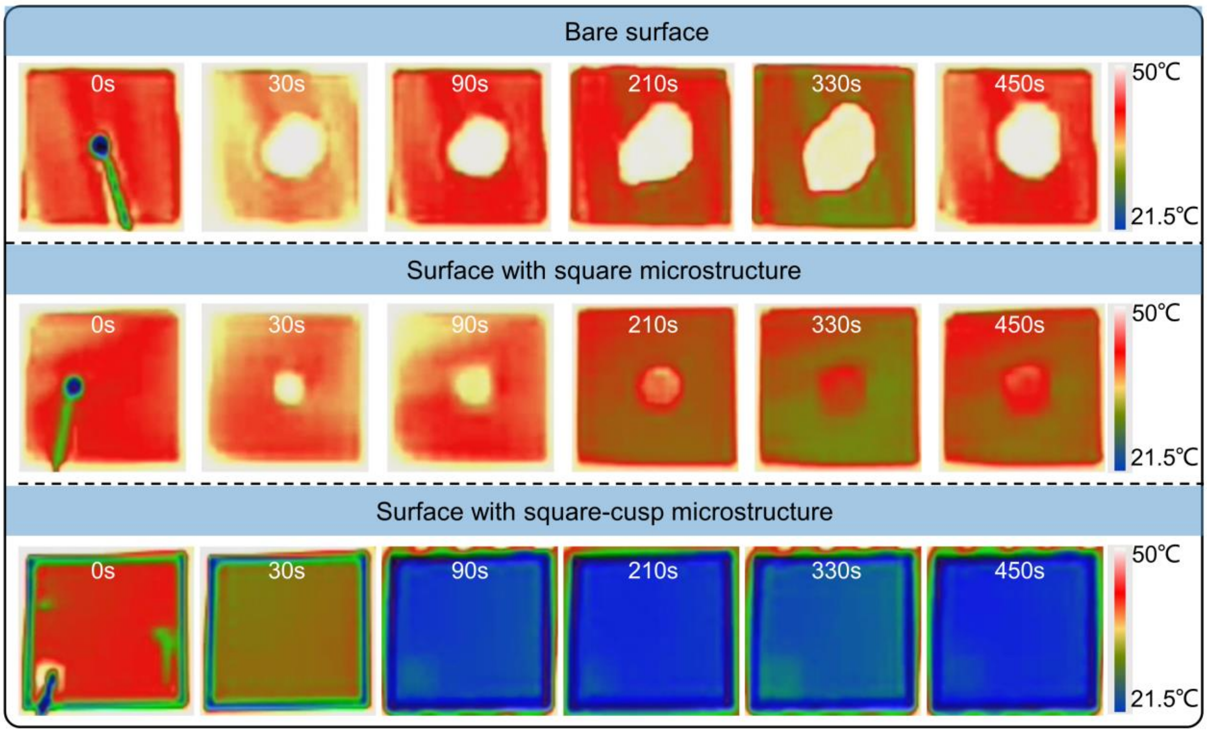

Fig. S37. Time-sequenced infrared thermal images under periodic deposition of 5 μL deionized water about every 30 s onto bare surface, square microstructured surface and square-cusp microstructured surface (S-mode II). The bare surface shows limited cooling with a small and transient cold region, while the square microstructured surface exhibits slightly enhanced cooling but with restricted spreading. In contrast, the square-cusp microstructured (S-mode II) surface enables rapid directional spreading and sustained evaporation, leading to a significantly enlarged and persistent cooling zone with surface temperature reduced toward about 24–26 °C.

Supplementary References

1. Jin L., et al. Friction mechanism of DLC/MAO wear-resistant coatings with porous surface texture constructed in-situ by micro-arc oxidation. *Surf. Coat. Technol.* **473**, 130010 (2023).
2. Lin P., et al. Effect and performance analysis of different surface treatments on polymer-metal friction pairs. *Tribol. Int.* **195**, 109602 (2024).
3. Dou X., et al. Self-dispersed crumpled graphene balls in oil for friction and wear reduction. *Proceedings of the National Academy of Sciences of the United States of America* **113**, 1528-1533 (2016).
4. Hou X.B., et al. Preparation and Tribological Properties of Graphene Lubricant Additives for Low-Sulfur Fuel by Dielectric Barrier Discharge Plasma-Assisted Ball Milling. *Processes* **9**, 272 (2021).
5. Karimi S., et al. Empirical investigation of the effect of adding nanoparticles to HB-80 gas turbine oil: Evaluation of thermophysical behaviors. *Heliyon* **10**, e29759 (2024).
6. Kumar M.S., et al. Enhancement in the Friction and Wear Resistance of Low Carbon Chromium Steel and Load Carrying Capability of MIL-PRF-23699 Grade Lubricant Using h-BN Nanoadditives for Aerospace Applications. *Tribology Transactions* **66**, 882-894 (2023).
7. Liu S.C., et al. Effects of Laser Surface Texturing and Lubrication on the Vibrational and Tribological Performance of Sliding Contact. *Lubricants* **10**, 10 (2022).
8. Cao H.Y., et al. Frictional Behaviour of the Microstructural Surfaces Created by Cylindrical Grinding Processes. *Applied Sciences-Basel* **12**, 618 (2022).
9. Chen K.P., et al. Enhancing Tribological Performance of Cylinder Guide Bush through Bionic Texturing and Composite Grease. *Tribology Transactions* **68**, 558-570 (2025).
10. Ge Z.H., et al. Surface Tribological Properties Enhancement Using Multivariate Linear Regression Optimization of Surface Micro-Texture. *Coatings* **14**, 1258 (2024).
11. Cai Q.S., et al. Influence of shot peening on the microstructure and friction-wear performance of CF53 steel. *Plos One* **20**, e0317410 (2025).
12. Zhang H., et al. Effect of Ultrasonic Rolling on Surface Properties of GCr15 Spherical Joint Bearing. *Lubricants* **12**, 208 (2024).
13. Hu G.L., et al. Effect of Low-Frequency Vibration on the Tribological Properties of Thin-Walled Inconel 601 with Laser Wire Additive Manufacturing. *Adv. Eng. Mater.* **27**, 2500187 (2025).

14. Acharya S., et al. Surface mechanical attrition treatment of low modulus Ti-Nb-Ta-O alloy for orthopedic applications. *Materials Science and Engineering: C* **110**, 110729 (2020).
15. Berce J., et al. Effect of Surface Wettability on Nanoparticle Deposition during Pool Boiling on Laser-Textured Copper Surfaces. *Nanomaterials* **14**, 311 (2024).
16. Orman L.J., et al. Analysis of Enhanced Pool Boiling Heat Transfer on Laser-Textured Surfaces. *Energies* **13**, 2700 (2020).
17. Orman L.J., et al. Laser Treatment of Surfaces for Pool Boiling Heat Transfer Enhancement. *Materials* **16**, 1365 (2023).
18. Zhao H.B., et al. Microstructured Ceramic-Coated Carbon Nanotube Surfaces for High Heat Flux Pool Boiling. *Acs Applied Nano Materials* **2**, 5538-5545 (2019).
19. Sen P., et al. Pool Boiling Performance on Cu-TiO_2 Nanoparticle-Coated Copper Surfaces Prepared Through Hybrid Method. *Heat Transfer Engineering* **46**, 345-361 (2025).
20. Gupta S.K., Misra R.D. Effect of two-step electrodeposited Cu-TiO_2 nanocomposite coating on pool boiling heat transfer performance. *J. Therm. Anal. Calorim.* **136**, 1781-1793 (2019).
21. Chun J., et al. Fast Capillary Wicking on Hierarchical Copper Nanowired Surfaces with Interconnected V-Grooves: Implications for Thermal Management. *Acs Applied Nano Materials* **4**, 5360-5371 (2021).
22. Luo J.L., et al. Biomimetic Copper Forest Wick Enables High Thermal Conductivity Ultrathin Heat Pipe. *ACS Nano* **15**, 6614-6621 (2021).
23. Luo J.L., et al. Biomimetic Copper Forest Structural Modification Enhances the Capillary Flow Characteristics of the Copper Mesh Wick. *Energies* **16**, 5348 (2023).
24. Wang J., et al. Enhanced ionic wind generation by graphene for LED heat dissipation. *International Journal of Energy Research* **43**, 3746-3755 (2019).
25. Xu C.L., et al. Enhanced Cooling of LED Filament Bulbs Using an Embedded Tri-Needle/Ring Ionic Wind Device. *Energies* **13**, 3008 (2020).
26. Cheng S.F., et al. On Electronics Cooling Using an Electrohydrodynamic Gas Pump with Aligned Electrodes. *J. Thermophys Heat Transfer* **38**, 360-367 (2024).
