# Supplementary material for: Bulk-cusp microstructure for controllable multi-directional liquid spreading
Source: Nat Commun. 2026 Jan 8;17:1519. doi: 10.1038/s41467-025-68237-8 (PMC12913607; doi:10.1038/s41467-025-68237-8)
Supplement: Supplementary file 2 — Description of Additional Supplementary Files [file 41467_2025_68237_MOESM2_ESM.pdf]

Description of Additional Supplementary Files

Supplementary Movie 1: Spreading behaviors of a droplet on the cross-cusp micro-structured surface in 5 array configurations (C-pinning and C-mode I–IV).

Description: The droplet body and precursor film exhibit distinct directional spreading or pinning depending on the cusp arrangement.

Supplementary Movie 2: Spreading behaviors of a droplet on the square-cusp micro-structured surface in 5 array configurations (S-pinning and S-mode I–IV).

Description: The precursor film spreads directionally while the droplet body remains pinned across all configurations.

Supplementary Movie 3: Droplet spreading on cross-cusp surfaces under continuous injection in four representative modes (C-mode I–IV).

Description: In C-mode I, the droplet spreads mainly along $y+$, in C-mode II along $x+$ and $y+$, in C-mode III along $x+$, $x-$, and $y+$, while in C-mode IV it spreads uniformly along $x+$, $x-$, $y+$, and $y-$ directions.

Supplementary Movie 4: Droplet spreading on square-cusp surfaces under continuous injection in four representative modes (S-mode I–IV).

Description: In S-mode I, the droplet spreads mainly along $y+$, in S-mode II along $x+$ and $y+$, in S-mode III along $x+$, $x-$, and $y+$, while in S-mode IV it spreads uniformly along $x+$, $x-$, $y+$, and $y-$ directions.

Supplementary Movie 5: High-speed snaps of fluid spreading on a C-mode II micro-structured surface.

Description: The precursor film initiates from the narrow gap between adjacent cusps and guides the directional spreading of the droplet body via asymmetric capillary forces.

Supplementary Movie 6: CFD simulation of time-resolved droplet spreading on cross-cusp microstructures in 5 representative modes (C-pinning, C-mode I–IV).

Description: Within the first 1 ms, droplets spread outward rapidly to form precursor films; from 5 to 10 ms, anisotropic patterns emerge, where C-mode I extends along *y*+, C-mode II and III spread toward *x*+ and *y*+ axis, and C-mode IV expands almost symmetrically in four directions, confirming the guiding effect of cusp geometry.

Supplementary Movie 7: CFD simulation of time-resolved droplet spreading on square-cusp microstructures in 5 representative modes (S-pinning, S-mode I–IV).

Description: From 0 to 1 ms, droplets spread outward rapidly, forming precursor films constrained by the square-cusp geometry. Between 5 and 10 ms, distinct spreading patterns emerge and stabilize, where S-mode I extends mainly along *y*+, S-mode II and III spread directionally toward multiple axes, and S-mode IV shows nearly symmetric expansion along four directions, highlighting the guiding role of the square-cusp structure.

Supplementary Movie 8: Time-dependent visualization of precursor film transport on the square-cusp micro-structured surface across S-mode I and S-mode II regions.

Description: The precursor film follows preset routes and undergoes turning at region boundaries, forming continuous wetting pathways.

Supplementary Movie 9: Infrared thermal imaging of the bare surface, square microstructured surface and square-cusp microstructured surface (S-mode II) after addition of a single 5 μL deionized water droplet.

Description: The microstructured surface shows rapid and uniform cooling, whereas cooling on the bare surface and square microstructured surface are limited and localized.

Supplementary Movie 10: Infrared thermal imaging of the bare surface, square microstructured surface and square-cusp microstructured surface (S-mode II) under repeated 5 μL water addition every 30 s.

Description: The square-cusp microstructured surface (S-mode II) maintains stable long-lasting cooling over time, in contrast to fluctuating thermal behavior and heat accumulation on the bare surface and square microstructured surface.
